# A single viral amino acid shapes the root system architecture of a plant host upon virus infection

**DOI:** 10.1186/s12866-024-03399-x

**Published:** 2024-07-19

**Authors:** Brandon G. Roy, Marc Fuchs

**Affiliations:** Plant Pathology and Plant-Microbe Biology Section, Cornell University, Cornell AgriTech at the New York State Agricultural Experiment Station, Geneva, NY 14456 USA

**Keywords:** Virus, Plant, Root architecture, Phenotype, Infection, Symptoms, Reactive oxygen species, Immune response

## Abstract

**Background:**

Grapevine fanleaf virus (GFLV) is one of the most detrimental viral pathogens of grapevines worldwide but no information is available on its effect on the root system architecture (RSA) of plant hosts. We used two wildtype GFLV strains and their single amino acid mutants to assess RSA traits in infected *Nicotiana benthamiana* and evaluate transcriptomic changes in host root gene expression in replicated time course 3’RNA-Seq experiments. Mutations targeted the multi-functional GFLV-encoded protein 1E^Pol*/Sd^, a putative RNA-dependent RNA polymerase and determinant of foliar symptoms in *N. benthamiana* plants.

**Results:**

Plant infection with wildtype GFLV strain GHu and mutant GFLV strain F13 1E^Pol^_G802K_, both carrying a lysine in position 802 of protein 1E^Pol*/Sd^, resulted in a significantly lower number of root tips (-30%), and a significantly increased average root diameter (+ 20%) at 17 days post inoculation (dpi) in comparison with roots of mock inoculated plants. In contrast, the RSA of plants infected with wildtype GFLV strain F13 and mutant GFLV strain GHu 1E^Pol^_K802G_, both carrying a glycine in position 802 of protein 1E^Pol*/Sd^, resembled that of mock inoculated plants. Modifications of RSA traits were not associated with GFLV titer. Root tissue transcriptome analysis at 17 dpi indicated dysregulation of pattern recognition receptors, plant hormones, RNA silencing, and genes related to the production of reactive oxygen species (ROS). For wildtype GFLV strain GHu, RSA modifications were correlated with an abundant accumulation of ROS in the pericycle of primary roots at 7 dpi and the duration of vein clearing symptom expression in apical leaves. Dysegulation of a hypersensitive response was an overarching gene ontology found through enrichment analyses of 3’RNA-Seq data.

**Conclusions:**

Our findings revealed the causative role of lysine in position 802 of protein 1E^Pol*/Sd^ in a novel RSA phenotype during viral infection and documented GFLV-*N. benthamiana* interactions at the root level based on (i) antiviral response, (ii) receptor mediated production of ROS, and (iii) hormone regulation. A correlation between above and below ground symptoms was reported for the first time in plants infected with wildtype GFLV strain GHu. Further work is warranted to test whether the modified RSA of a plant host might impact GFLV acquisition and transmission by the ectoparasitic dagger nematode *Xiphinema index*.

**Supplementary Information:**

The online version contains supplementary material available at 10.1186/s12866-024-03399-x.

## Introduction

Roots play a crucial role in plant development, contributing largely to overall health and growth. Plants rely on this underground network of tissue to sequester nutrients, acquire water, provide energy sinks for long chain carbohydrates, and act as anchorage to combat adverse environmental stressors. The configuration of a root system in the soil is commonly referred to as root system architecture (RSA). The overall RSA is dynamically influenced by abiotic and biotic factors, including pathogens such as viruses that can negatively modify RSA upon infection of plant hosts [1–3].

Some plant viruses are transmitted by soil-borne vectors [4] and are known to manipulate host root systems. For example, tobacco rattle virus (TRV) alters RSA and root volatiles of *Nicotiana benthamiana*, affecting transmission characteristics by trichodorid nematode vectors [5]. TRV manipulates the production pathway of 2-ethyl-1-hexanol to attract nematodes for feeding on infected tissue, and subsequentially to increase virus acquisition for more efficient transmission. Similarly, the RNA3-encoded p25 protein of beet necrotic yellow vein virus (BNYVV) determines both foliar and root symptoms in sugar beet (*Beta vulgaris* subsp. *vulgaris*) [6]. The constitutive expression of BNYVV protein p25 in *Arabidopsis thaliana* induces hormonal changes and a distinct RSA phenotype [1].

Grapevine fanleaf virus (GFLV, species *Nepovirus foliumflabelli*, genus *Nepovirus*, family *Secoviridae,* order *Picornavirales*) is a causative agent of fanleaf degeneration disease, and the most detrimental viral pathogen of grapevines worldwide [7]. In the vineyard, GFLV is transmitted by the ectoparasitic dagger nematode *Xiphinema index* in an exclusive manner, solely dependent upon the viral coat protein [8–10]. The GFLV genome is bipartite with two single-stranded RNA molecules (RNA1 and RNA2). GFLV RNA1 encodes proteins involved in polyprotein processing [11], replication [12, 13], and suppression of RNA silencing [14], while GFLV RNA2 encodes proteins involved in replication [15], cell-to-cell movement [16], and encapsidation [17]. Performing experiments with GFLV in the natural host (*Vitis* spp.) can be laborious and time-consuming; therefore, herbaceous model hosts are commonly utilized for GFLV-plant interaction studies [18]. Among herbaceous hosts, *N. benthamiana* is frequently used to study virus-plant interactions because it can easily be infected via mechanically inoculation or through *Agrobacterium tumefaciens*-mediated infiltration of GFLV infectious clones, among other factors [19].

Previous work revealed a single amino acid of GFLV strain GHu is responsible for a transient vein clearing phenotype in apical leaves of *N. benthamiana* plants [20, 21]. This symptom determinant (Sd) residue is located at position 802 (or position nts 2,404–2,406) of the GFLV RNA1-encoded putative RNA-dependent RNA polymerase (Pol*), refered to as protein 1E^Pol*/Sd^ [20–22]. GFLV strain GHu encodes a lysine (K) at position 802 of protein 1E^Pol*/Sd^ which is necessary but not sufficient to produce these symptoms in GFLV strain F13 [20, 21]. The viral symptom determinant region is hypothesized to interact with host components that ultimately elicit or repress an immune response, although structural or functional homologues could not be identified by advanced protein modeling techniques [22]. A combined time-resolved transcriptomic and proteomic approach documented a reactive oxygen species (ROS) molecular pathway in addition to the dysregulation of transcripts and proteins of plant immune response, transcriptional regulation, and translational regulation in *N. benthamiana* leaves upon GFLV infection [23]. Transient leaf symptoms can be observed on leaves of *Vitis* and *Nicotiana* species alike. Although experimental evidence of ROS production in *N. benthamiana* following GFLV infection is lacking, other studies have correlated ROS production with pronounced symptoms in GFLV-infected *Vitis* cultivars [24] or detected ROS in GFLV-infected *N. occidentalis* [25].

Studies on GFLV-host interactions have centered on above-ground plant tissue to investigate underpinnings of symptom development in leaves and determine how fruit yield and quality are affected by viral infection [7, 16, 19–21, 23–25]. No research has investigated the effects of GFLV on the RSA of a plant host. The aim of this study was to explore below ground features of *N. benthamiana* plants and probe the molecular dysregulation of root tissue upon GFLV infection. We hypothesized that RSA traits follow similar phenotypic trends to foliar symptomology, and that GFLV elicits similar transcriptomic profiles in roots as previously observed with leaves [19]. Here, we document GFLV protein 1E^Pol*/Sd^ single amino acid-specific modifications of RSA traits, ROS production in roots, and root transcriptome dysregulation upon infection.

## Materials and methods

### *Nicotiana benthamiana* plants

Seeds of wildtype *N. benthamiana* were sown in Cornell LM-2 soil, transplanted ~ 14 days after germination, and maintained in a controlled growth chamber at 25 °C and 80% humidity, as previously documented [19]. Plants were watered daily and fertilized (Scotts Miracle-Gro® All Purpose Plant Food 24–8-16) weekly. Four independent experiments were performed with 75, 74, 107, and 80 plants referred to as cohorts 1–4, respectively.

### GFLV strains

Four GFLV strains were selected for this study: wildtype GFLV strains GHu and F13, and their mutants carrying a single amino acid change at position 802 of the RNA1-encoded RNA-dependent RNA polymerase [21]. These strains will be referred herein to as wildtype GHu, wildtype F13, mutant GHu 1E^Pol*/Sd^_K802G_, and mutant F13 1E^Pol*/Sd^_G802K_.

### Plant mechanical inoculation with GFLV strains and leaf phenotyping

The genome of GFLV strains used in this study consisted of RNA1 of wildtype F13, wildtype GHu, mutant F13-1E^Pol*/Sd^_G802K_, or mutant GHu-1E^Pol*/Sd^_K802G_, alongside RNA2 of GFLV-GHu [19]. These combinations of GFLV RNA1 and RNA2 were selected for their high infection rates in *N. benthamiana* [21]. Infected plant tissue stored at -80 °C was used for mechanical inoculations [20]. The infection status of inoculated plants was confirmed in apical uninoculated leaves by double antibody sandwich enzyme-linked immunoassay (DAS-ELISA) at 14 days post inoculation (dpi) using GFLV specific antibodies (BIOREBA, Switzerland) (19). At 4 dpi, the status of GFLV infection was tested in inoculated leaves through RT-PCR and in roots by both RT-qPCR and 3’RNA-Seq of total RNA.

Leaves of *N. benthamiana* plants inoculated with wildtype GHu were rated daily for vein clearing symptoms on a binary scale: 0 for asymptomatic, and 1 for symptomatic. No symptoms were observed on the other GFLV treatments or mock control plants, as previously reported [21, 23].

### Root system excavation, image acquisition and processing, and root trait analysis

The root excavation pipeline used in this study was previously reported as an open access BioProtocol [26]. Briefly, potted plants were gently released of soil by hand and root systems were dipped in water until all soil was removed. If persistent soil was adhering to roots, a spray bottle of distilled water was used to remove excess debris. The roots were dried on a paper towel and immediately placed on clear plastic imaging trays [26].

Imaging trays were placed on the scanning bed of an Epson Expression XL12000 photo scanner (Model:12000XL-PH) connected to a computer for image acquisition of the roots with the following parameters: ≥ 400 dpi, grayscale, and best quality. Images were analyzed in RhizoVision Explorer version 2.0.3 [27] with the following parameters: analysis mode (Broken roots), pixels converted to physical units at ≥ 400 dpi, image thresholding level = 158, (158 for cohort 1, 186 for cohort 2, 182 for cohort 3) (manually adjusted per image ± 10), filter non-root objects, enable edge smoothing = 2, feature extraction levels by root diameter (in pixel units) for fine (0.00 – 0.50), small (0.50 – 0.75), medium (0.75 – 1.75), and large (more than 1.75) roots. Using dpi and conversion factors of image acquisition, these measurements corresponded to thresholds of 0.00 mm, 0.32 mm, 0.48 mm, and 1.11 mm. Ultra-fine roots were not considered in this study because the excavation pipeline was optimized to analyze crown root tissue only pertaining to the first several orders of the root system including the primary root with secondary, and at least tertiary lateral roots extending off the primary. Output data was transferred to a Microsoft Excel file. All raw data, including images, are available on the GitHub page or within the supporting information.

### Measurement of *N. benthamiana* plant height and biomass

The height of *N. benthamiana* plants was measured from the soil line barrier to the apical most part of the central stem with a ruler one day prior to root excavation. Shoots of *N. benthamiana* plants were collected into brown paper bags and thereafter roots were placed in the corresponding numbered bag. Dry biomass was measured after ~ 1 week in a drying oven at 105 °C. The ratio of root to shoot biomass was calculated for select cohort of *N. benthamiana* plants to assess biomass allocation.

### 3, 3'-diaminobenzidine (DAB) staining of *N. benthamiana* primary roots

Primary root samples of *N. benthamiana* plants were excavated from soil at 7- and 11-dpi for histological staining. For this, five plants per treatment were sacrificed for tissue collection, where at least three or more of the plants tested true to infection status through RT-PCR. Roots were vacuum infiltrated with 1 mg/mL DAB solution for five minutes and kept in the dark at ambient conditions overnight (8–12 h) [28]. De-staining was not performed because there is no chlorophyll to extract, and roots produce high amounts of ROS when exposed to ethanol or other abiotic stressors. Cross-sections of roots were made by hand just above where lateral roots formed (e.g., first branching points of root system) and performed two centimeters from the cut made at the stem of plants during excavation. Root cross-sections were placed on a glass microscope slide (Fisher, Cat No. 12–550-A3) mounted with 0.1 mL of 50:50 v/v glycerol:PBST (137 mM NaCl, 2.7 mM KCl, 10 mM Na_2_HPO_4_, 1.8 mM KH_2_PO_4_), and protected with a coverslip (Fisher, Cat No. 12–545-88). Imaging of root cross sections was performed immediately following slide mounting to ensure no supplemental induction of ROS production due to additional stress. Images were acquired on an Olympus SZX12 microscope using the following parameters: Gamma 1.16, Contrast 0.79, Saturation 1.53, Color Temp 4188, Exposure 160–200, Gain 1.

### *N. benthamiana* root tissue collection, nucleic acid extractions, RT-PCR, and RT-qPCR

After root system images were acquired, five *N. benthamiana* plants that tested true to virus infection status were randomly chosen for GFLV titer analysis. Roots of mock inoculated *N. benthamiana* plants were used as controls in quantification assays. Approximately half of the root system was sectioned by excising primary, lateral, and fine roots with a sterile razor blade. Samples were placed in 2 mL Eppendorf tubes, flash frozen in liquid nitrogen, and stored at -80 °C until further processing. Total nucleic acid was extracted from root samples using E.Z.N.A.® Plant RNA Kit (SKU:R6827-01) with a final elution volume of 120 µL nuclease-free water. Then, 40 µL of the eluted nucleic acids were used for DNAse I treatment at 20 °C for 15 min followed by RNA clean-up with Zymo Research RNA Clean & Concentrator®-5 (Cat # R1013). RNA integrity and quantity was assessed on an Agilent 2100 Bioanalyzer with the RNA 6000 Nano Kit Assay (No. 5067–1511) according to manufacturer instructions.

Total RNA was used to amplify the GFLV RNA1 nucleotide sequence region covering amino acid in position 802 of protein 1E^Pol*/Sd^ with the QIAGEN® OneStep RT-PCR Kit (No. 210210) for Sanger sequence confirmation, as previously described [21]. Total RNA was also used for GFLV titer analysis by Luna® Universal One-Step RT-qPCR (Cat. No. E3005) with primers specific to GFLV RNA1 [21] and to the endogenous *N. benthamiana* control FBOX gene [29] (Supplementary Table 1). Samples were tested in triplicate, and quantification cycle (Cq) values for SYBR green were collected after each cycle, as previously reported [21]. GFLV treatments were compared with mock inoculated control samples to calculate ∆∆Cq values, in which relative expression was calculated as log(2^−∆∆Cq^) values [30].

### GFLV strain identification

Amplified RT-qPCR products of ~ 250 bp in size obtained with GFLV RNA1 primers from roots of two to four infected plants at 17- and 26 dpi were cleaned up with Zymo DNA Clean & Concentrator®-5 (Cat #D4004). The integrity of the two wildtype and two mutant GFLV strains was tested in roots through Sanger sequencing of the cleaned amplicons. Sequences obtained in this study were compared with GFLV sequences deposited in GenBank (NC003615 for wildtype F13 RNA1 and JN391442 for wildtype GHu RNA1) using SeqMan Ultra™ (v.17.3.1) of the DNASTAR Navigator Package (v17.3.0.57) (DNASTAR, Inc., Madison, Wisconsin, USA).

### 3 ‘-ribonucleic acid sequencing

The transcriptome of *N. benthamiana* roots was characterized via 3’RNA-Seq for wildtype GHu, mutant GHu 1E^Pol*/Sd^_K802G_, and mock control with samples collected at 4-, 17-, and 26 dpi. Three biological replicates for each time point per viral treatment were purified, concentrated, and DNAse I treated to produce a total of 27 samples. Libraries were prepared with the Illumina® TruSeq RNA Library Prep Kit (RS-122-200X) and sequenced via Illumina® NextSeq500/550. Transcript reads were trimmed with Trimmomatic (v0.39) [31] and subsequently mapped to the host genome, as well as the previously curated GFLV library ‘Nepo’ [23] using HiSat2 (v2.2.1) [32]. Sequences were checked for quality control using the FastQC program (v0.11.9) [33].

### Bioinformatic analysis

Sequence reads were mapped to the *N. benthamiana* genome from two different sources: Niben v1.0.1 (Boyce Thompson Institute, Ithaca, NY, USA) [34] with 61,328 gene models and Nbe_v1 [35] with 57,583 high confidence gene models. Through a bioinformatic pipeline, the Niben v1.0.1 genome was reannotated in this study using an updated list of gene ontology identifiers by subjecting all protein encoding sequences to a BLAST search through EggNOG (v5.0) [36] [Supplementary Fig. 1]. After curation of additional annotation terms, the files were parsed and linked through the GO API (Open Biological and Biomedical Ontology Foundry, http://api.geneontology.org/api) to create a .gmt file for use in gene enrichment. Data files were then manually curated in RStudio and R [37, 38].

The DESeq2 package (v1.40.2) [39] was used to normalize, test differential transcript abundance, and prepare data for downstream analysis, as performed previously [23]. Gene set enrichment analysis was performed using gProfiler (v0.2.1) [40] and clusterProfiler (v4.8.2) [41] packages. Additional packages used for visualization and data analysis include the following: enrichplot (v1.20.1) [42], pathview (v1.40.0) [43], dplyr (v1.3.1) [44], ggplot2 (v3.4.3) [45], and GO.db (v3.16.0) [46].

### Statistical analyses

Matrices of acquired root traits were subject to Pearson’s correlation analysis [47] with the hierarchal clustering method. RStudio package corrplot (v0.92) [48] was employed to calculate correlation coefficients (-1 to + 1) and p-values at four significance intervals (* *p* < 0.05, ** *p* < 0.01, *** *p* < 0.001, **** *p* < 0.0001). In the instances of parametric data and normal distribution, root data were analyzed through analysis of variance (ANOVA) followed by student’s t-test for mean testing differences amongst treatments to controls. The p-value for parametric data is reported through the adjustment of Holm’s method. For instances of data with non-parametric or non-normal distributions, Kruskal–Wallis [49] tests followed by a Bonferroni correction [50] were performed. Tukey’s HSD with associated letters of significance were utilized for follow up statistical testing in root phenotype analyses. A Dunn’s test with Holm’s method adjusted p-values was utilized for mean comparison testing in non-parametric data. For the analysis of virus titer by RT-qPCR, logarithmic transformed values were used in a geometric mean comparison [51] and multiple t-test. Further analysis of RNA-Seq data utilized native statistical testing within clusterProfiler, DESeq2, WGCNA, enrichplot, and gProfiler packages.

Days showing symptoms (DSS) on apical leaves of *N. benthamiana* plants infected with wildtype GHu were calculated by subtracting the first and last day of symptom observation, and this metric was used as a predictor variable for RSA traits. Subsequent linear models were constructed and visualized in RStudio through the intrinsic stats package (v4.1.3). Mixed models were also constructed by using the predictors of day first symptomatic (DFS), day last symptomatic (DLS), and the interaction of these two terms with regards to foliar symptoms of wildtype GHu.

## Results

### Confirmation of GFLV infection status and strain integrity in N. benthamiana plants

At 4 dpi, GFLV was detected in most inoculated leaf samples of representative infected plants by RT-qPCR (75%, 15/20) or by RNA-Seq of root tissue (100%, 6/6), but was not detectable by DAS-ELISA, as expected for such an early infection stage. At 14 dpi, a high GFLV infection rate was observed in three cohorts of plants [100% (60/60) for cohort #1, 73% (52/71) for cohort #2, and 91% (85/93) for cohort #3] in apical leaves through DAS-ELISA. None (0%, 0/38) of the mock inoculated control plants tested positive for GFLV in DAS-ELISA in any of the replicated experiments, as expected.

A glycine (GCC in RNA1 nt position 6,787–6,789) was found in the progeny of wildtype F13 and mutant GHu 1E^Pol*/Sd^_K802G_, while a lysine (AAG in RNA1 nt position 6,797–6,799) was observed in the progeny of wildtype GHu and mutant F13 1E^Pol*/Sd^_G802K_, as anticipated (Supplementary Table 1). These analyses revealed high GFLV infection rates of inoculated *N. benthamiana* plants, and the genetic integrity of the GFLV progeny (Supplementary Table 2).

### RSA trait acquisition and analysis

**Fig. 1:**
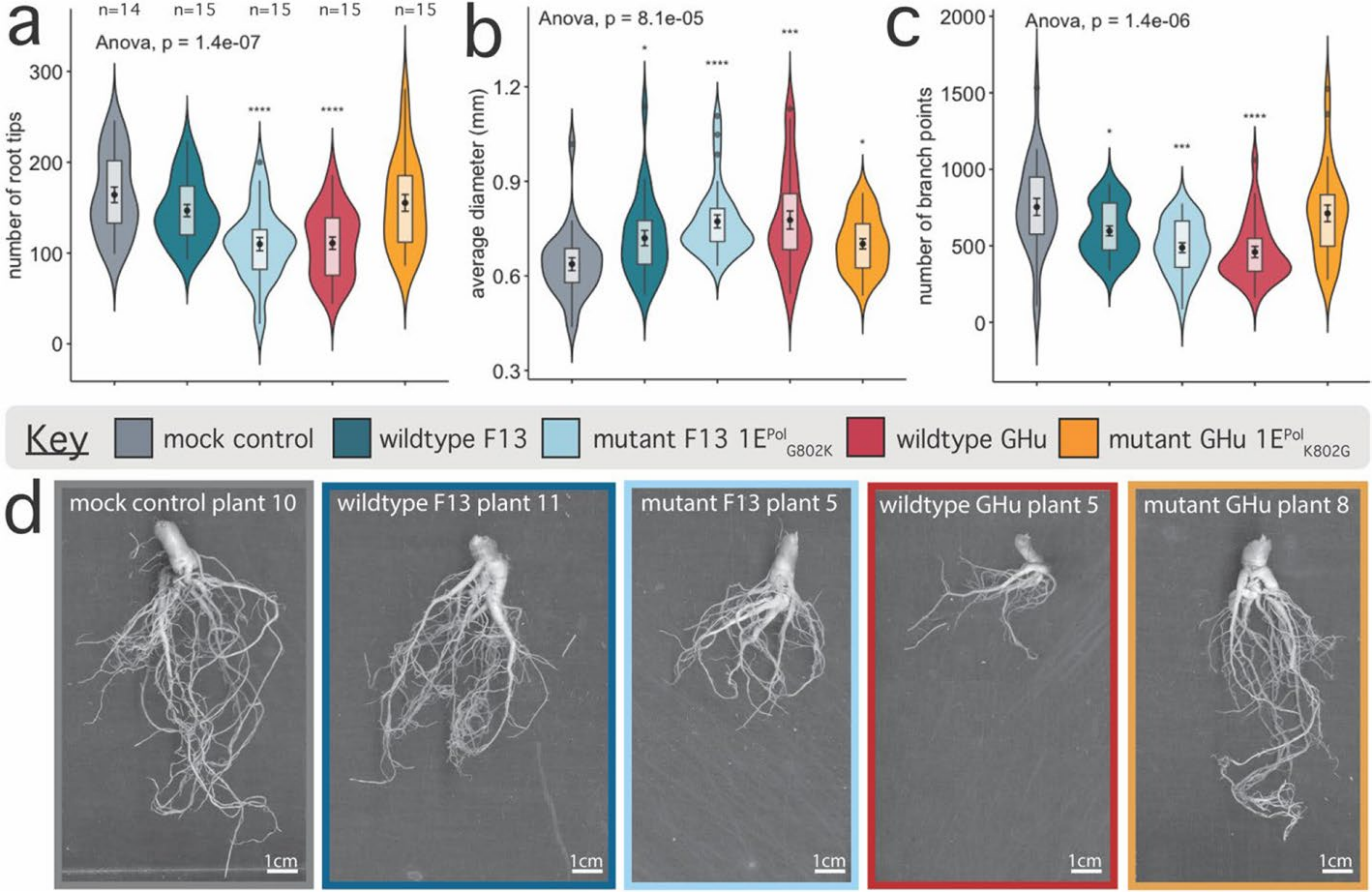
Root system architecture traits for *Nicotiana benthamiana* infected with four strains of grapevine fanleaf virus (i.e., wildtypes GHu and F13, and mutants GHu 1E^Pol*/Sd^_K802G_ and F13 1E.^Pol*/Sd^_G802K_) at 17 days post-inoculation. **a** Number of root tips per plant, **(b)** average root diameter in millimeters, and **(c)** number of branching points per treatment group. Distribution of each continuous value is plotted along the y-axis as a violin plot with boxplot within, showing additional mean with standard error (central point with error bars). ANOVA significance values are displayed in top left of each panel, as well as students t-test relative to control as significance values (< 0.05 = *, < 0.01 = **, < 0.001 = ***, < 0.0001 = ****). The number of samples per treatment is displayed above panel A. **d** Representative images of mean phenotype observed between treatments with border color indicative of treatment as in plots A, B, and C. White bar displayed in bottom right corner of each image is 1.0 cm, calculated in ImageJ software (v1.8.0.172)

**Fig. 2:**
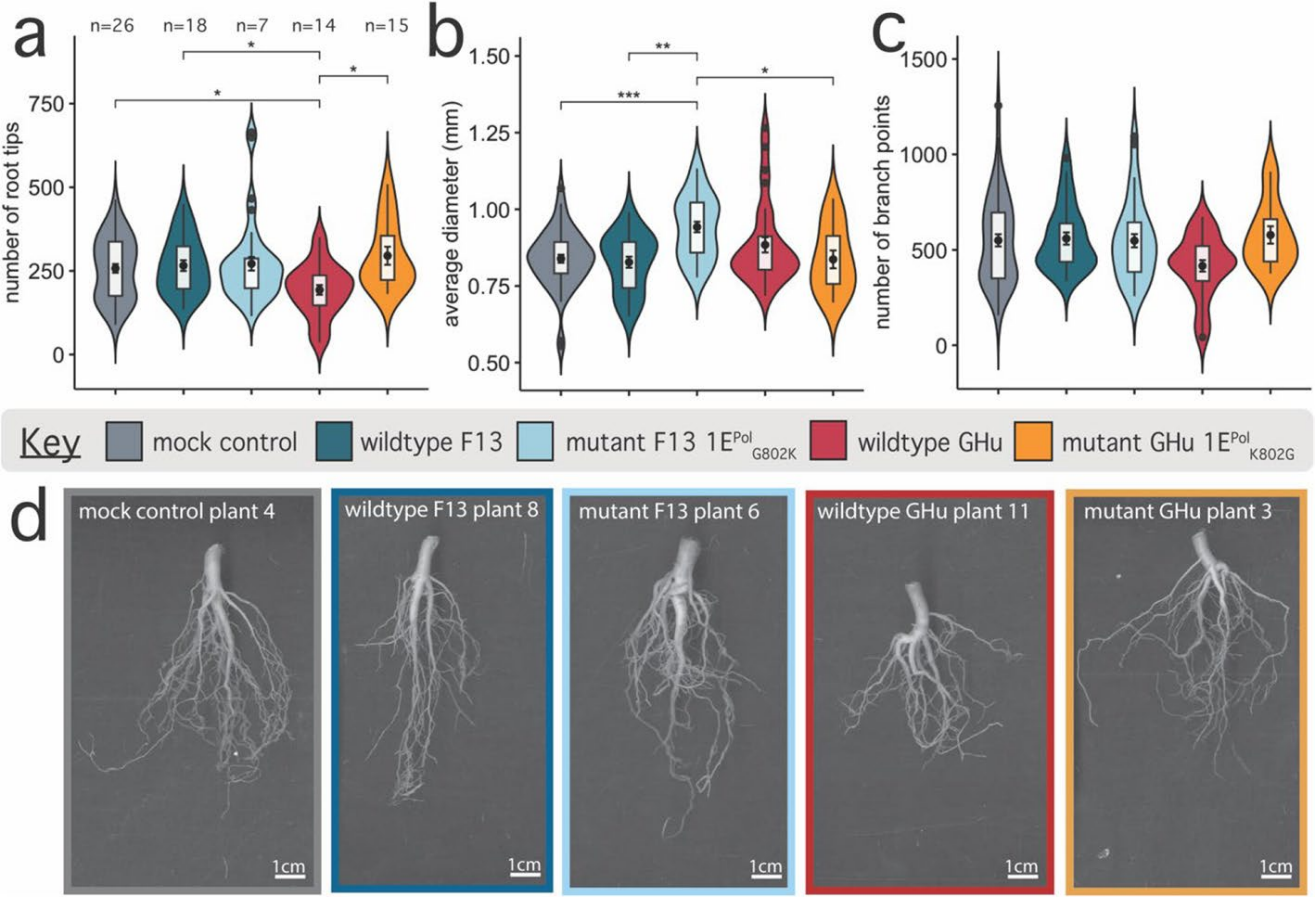
Root system architecture traits for *Nicotiana benthamiana* infected with four strains of grapevine fanleaf virus (i.e., wildtypes GHu and F13, and mutants GHu 1E^Pol*/Sd^_K802G_ and F13 1E.^Pol*/Sd^_G802K_) at 26 days post-inoculation. **a** Number of root tips per plant, **(b)** average diameter in millimeters, and **(c)** number of branch points plotted per viral treatment group. Distribution of each continuous value is plotted along the y-axis as a violin plot with boxplot within, showing additional mean with standard error (central point with error bars). Pairwise Wilcox test results are displayed as significance values (< 0.05 = *, < 0.01 = **, < 0.001 = ***, < 0.0001 = ****). The number of samples per treatment is displayed above panel A. **d** Representative images of mean phenotype observed between treatments, border color is indicative of treatment as in plots A, B, and C. White bar displayed in bottom right corner of each image is 1.0 cm, calculated in ImageJ software (v1.8.0.172)

Roots of GFLV-infected and mock inoculated *N. benthamiana* plants were excavated to result in 263 crown root samples being scanned at ≥ 400 DPI. A total of 77 images captured a first and second side of the whole RSA per plant after a 180º rotation. The three time points of excavation, i.e., 4-, 17-, and 26 dpi, displayed distinct phenotypes with the most dramatic at 17 dpi for plants infected with wildtype GHu (Figs. 1 and 2, Supplementary Fig. 2).

**Fig. 3:**
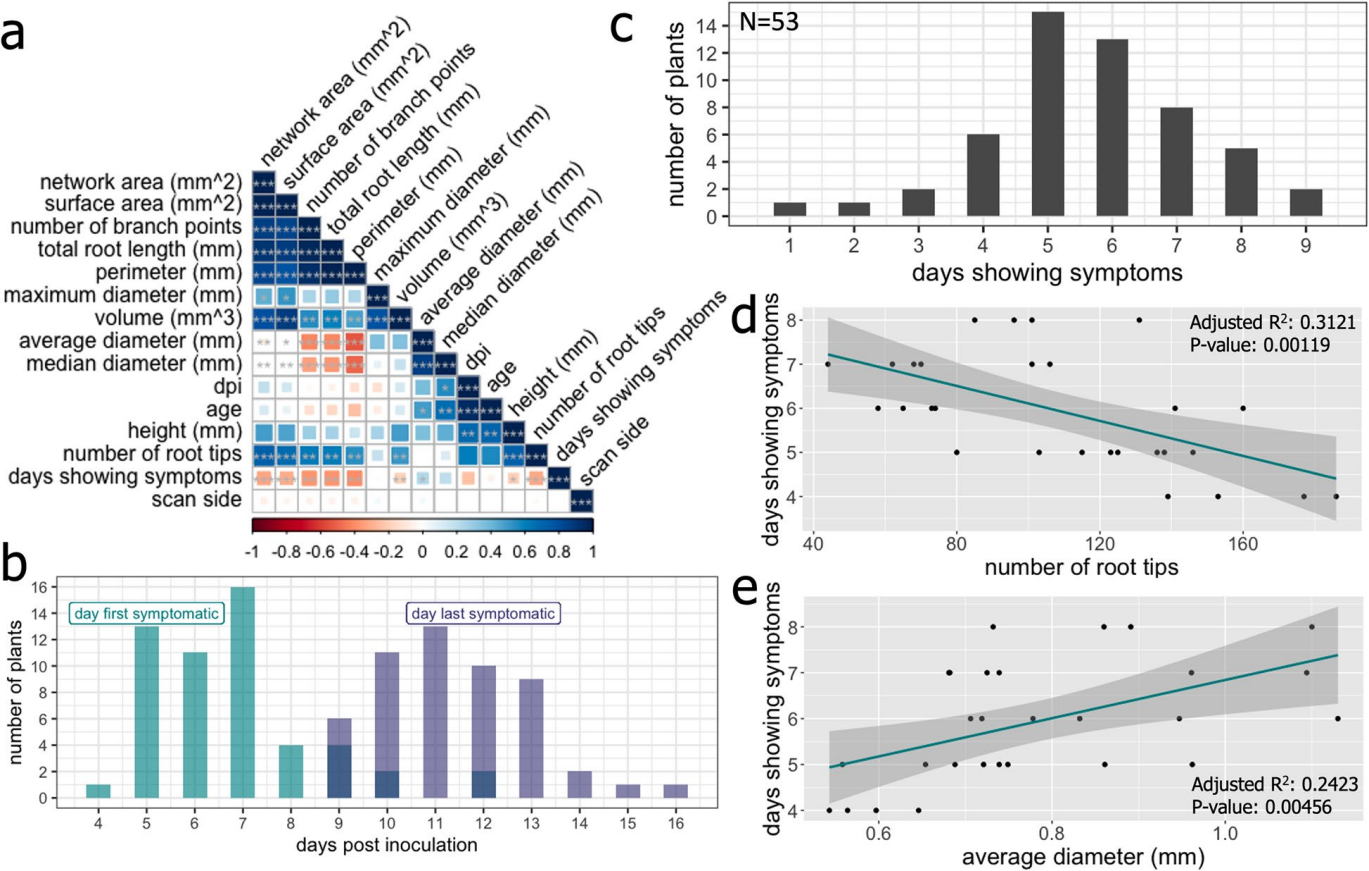
The root system architecture (RSA) of *Nicotiana benthamiana* plants upon grapevine fanleaf virus infection is dependent on the identity of amino acid 802 in protein 1E^Pol*/Sd^. **a** RSA traits across four experimental replicates show highly correlated phenes that are dependent upon one another across all treatment groups. The variable ‘days showing symptoms’ was calculated from observational documentation of vein clearing symptoms in apical leaves for wildtype GHu infected plants only. **b** A histogram displays the first and last day of symptoms (in teal and purple) before full recovery for apical leaves in wildtype GHu infected *N. benthamiana*. **c** A normal distribution appears when calculating the total days symptomatic across three experimental replicates (*n* = 56). At 17 days post inoculation **(d)** the number of days showing symptoms had a negative relationship with the number of total root tips and inversely, **(e)** the days showing symptoms in leaves had a positive relationship with the average diameter in millimeters

By focusing on ten root traits (number of root tips, number of branch points, total root length, branching frequency, network area, average diameter, median diameter, perimeter, volume, and surface area) of the 32 metrics obtained by Rhizovision analysis, significant differences were found with several co-variable or often highly correlated traits (Fig. 3a). Five root traits (number of root tips, root diameter, total root surface area, and root branching frequency) were selected for further analysis through pairwise treatment testing at each time point.

At 4 dpi, all GFLV strains indicated some level of initial RSA perturbation (Supplementary Fig. 4d) with an overall 7–23% reduction in root tip number, a 20–25% increase in average diameter, and a 30–48% reduction in branching points, when compared with mock inoculated plants. However, no significant differences were observed for the number of root tips for virus strain, while the average diameter significantly differed between all viral strains compared to mock controls, as shown by Tukey’s test and pairwise analysis (Supplementary Fig. 4a,b). The number of branching points was less conclusive because all viral treatments, except wildtype GHu, were significantly different from mock inoculated plants, and wildtype GHu overlapped with all treatments according to Tukey’s test (Supplementary Fig. 4c). The limited changes in the overall RSA at 4dpi were likely due to younger plants being exposed to the effects of viral infection for a short period.

At 17 dpi, wildtype GHu and mutant F13 1E^Pol*/Sd^_G802K_ caused up to a 33% reduction of the total number of root tips (****p < 0.0001, Fig. 1a), and a 21%-22% increase in average root diameter compared with mock inoculated control plants (***p < 0.001, Fig. 1b, Supplementary Tables 3, 4). These two GFLV strains also decreased root branching frequency, a measure of the number of branch points per total root length by up to 10.1% (**p < 0.01, Fig. 1c, Supplementary Tables 3, 4). Reduced root tip numbers, less fibrous but thicker roots were visually apparent for wildtype GHu and mutant F13 1E^Pol*/Sd^_G802K_ compared with the other treatments (Fig. 1d). Wildtype F13 and mutant GHu 1E^Pol*/Sd^_K802G_ increased the average root diameter by 13% and 10%, respectively, compared with mock inoculated control plants (*p < 0.05, Fig. 1d). Dry root biomass was reduced in plants infected wildtype GHu (21%), wildtype F13 (9%), and mutant GHu 1E^Pol*/Sd^_K802G_ (10%) when compared with mock controls while plants infected with mutant F13 1E^Pol*/Sd^_G802K_ had the same biomass as mock controls (Supplementary Fig. 5). These results documented drastic changes in the RSA of plants infected with wildtype GHu and mutant F13 1E^Pol*/Sd^_G802K_, both containing K at position 802 of protein 1E^Pol*/Sd^, at 17 dpi.

At 26 dpi, plants infected with wildtype GHu exhibited a 25% reduction in the total number of root tips (*p < 0.05) and a 6.0% increase in average root diameter when compared with mock control plants (Fig. 2a, b, Supplementary Tables 3, 4). Plants infected with mutant F13 1E^Pol*/Sd^_G802K_ displayed an increased average root diameter of 13% (***p < 0.001) but little change in the number of root tips (*p* > 0.05) when compared with mock control plants. Branching frequency was consistent across all treatment groups (Fig. 2c). These characteristic RSA traits were apparent on pictures of representative root systems (Fig. 2d). Dry root and dry shoot biomass decreased by 26% and 20%, respectively, for plants infected with wildtype GHu, while an increase in these metrics was observed with all other virus strains in comparison with mock inoculated plants (Supplementary Table 5). These results documented drastic changes in the RSA of plants infected with wildtype GHu and to a lesser extent with mutant F13 1E^Pol*/Sd^_G802K_, both containing K at position 802 of protein 1E^Pol*/Sd^, at 26 dpi. Additionally, many traits such as perimeter, surface area, root to shoot biomass, and plant height were less pronounced when comparing treatments at all time points (Supplementary Fig. 5).

### GFLV titer in root tissue

No significant differences in GFLV titer were found at 4 and 17 dpi, as shown by student’s t-test of RT-qPCR data (Supplementary Fig. 6a). At 26 dpi, the titer of wildtype F13 was significantly higher from both wildtype GHu and mutant GHu 1E^Pol*/Sd^_K802G_, however no other viral treatments differed through student’s t-test (Supplementary Fig. 6a). The titer of wildtype F13 was significantly different between 17- and 26-dpi, as shown by a Wilcoxon test (*p = 0.016), however no other viral strain had significantly different titer between time points (*p* > 0.05) (Supplementary Fig. 6a). As expected, mock control plants retained no viral titer. These data suggested that virus titer was unrelated to RSA traits during symptom development.

To complement RT-qPCR data, GFLV RNA1 and RNA2 reads from 3’RNA-Seq data were extracted and analyzed. No significant differences were observed amongst viral strains at 4 dpi or 17 dpi, but highly significant differences in RNA1 (**p = 0.00014) and RNA2 (****p = 0.0000008) were observed when comparing wildtype GHu to mutant GHu 1E^Pol*/Sd^_K802G_ at 26 dpi, as shown by Tukey’s HSD test (Supplementary Fig. 6b,c). This trend was numerically similar but not statistically significant in the RT-qPCR data. Together, these data suggested that GFLV titer is not directly correlated with root phenology at 17 dpi but differed amongst viral strains and corresponding root phenotype later in infection.

### Effect of GFLV infection on plant height

At 4 dpi, plants infected with wildtype GHu and F13 had a 2% and 6% decrease in height, respectively, while plants infected with mutant GHu 1E^Pol*/Sd^_K802G_ and F13 1E^Pol*/Sd^_G802K_ displayed a 1% and 9% increase in height, respectively (Supplementary Table 5, Supplementary Fig. 5). At 17 dpi, GFLV-infected plants had increases in height (up to 18%) for asymptomatic strains and symptomatic wildtype GHu (up to 3%) (Supplementary Table 5, Supplementary Fig. 5). At 26 dpi, height was reduced by an average of 16% and 9% for plants infected with wildtype GHu and F13, while mutant GFLV strains displayed no change or a minor increase in height when compared with mock inoculated plants (Supplementary Fig. 5). These data revealed that GFLV infection had overall more drastic effects on plant height at late infection stages rather than during early infection.

### Correlation of RSA traits and foliar symptomatology for wildtype GHu

A strong positive correlation was observed for root diameter and days showing symptoms (DSS) in apical leaves. Conversely, a strong negative correlation was found between DSS and root branching traits such as root tip number, number of branching points, and network area (Fig. 3a, Supplementary Fig. 3). At 17 dpi, dry biomass, network area, perimeter, and surface area were significantly reduced for plants infected with wildtype GHu, and to lesser extents across other viral strains several, illustrating modifications above and below ground (Supplementary Table 5). At 26 dpi, additional traits were significantly impacted but not to the level described at 17 dpi (Supplementary Table 5). While plants infected with other viral strains remained close to mock control plants, wildtype GHu induced a lessened amount of root tips, number of branch points, total root length (mm), network area (mm^2^), perimeter (mm), volume (mm^3^), and surface area (mm^2^) (Supplementary Table 5).

Since many RSA traits were significantly and strongly correlated (positively or negatively) with leaf symptomology in *N. benthamiana* infected with wildtype GHu, general linear models and mixed models were constructed to predict RSA traits. A relatively normal distribution of symptom emergence centered at 7 dpi (average 6.77 dpi) with symptom disappearance around 11 dpi (average 11.37 dpi) and vein clearing symptoms apparent for an average of 5.66 days across all 53 *N. benthamiana* plants infected by wildtype GHu (Fig. 3b,c, Supplementary Table 6). A linear model constructed using the continuous response variables from acquired RSA traits against the predictor variable of days symptomatic documented an inverse relationship between the number of root tips and the number of days symptomatic in leaf tissue (RSE = 32.35, adjusted R^2^ = 0.254, *p* = 0.00266, Fig. 3d). The observed variance in average root diameter could be significantly explained by the symptomatic days (RSE = 0.1454, adjusted R^2^ = 0.1517, *p* = 0.01912, Fig. 3e), albeit explaining less variation compared with root tip number. These results illustrated an association between symptoms above and below ground for *N. benthamiana* infected with wildtype GHu.

A mixed model incorporating DFS and DLS as date component rather than difference of the two dates performed nearly twice as well for modeling root tip number (RSE = 23.95, adjusted R^2^ = 0.6067, *p* = 1.105E-5), and nearly as well for median root diameter prediction (RSE = 0.07733, adjusted R^2^ = 0.3649, *p* = 0.002929). At 26 dpi, correlation analysis was strong although linear modeling returned insignificant results [p > 0.05] (Supplementary Fig. 7). These observations suggested a correlation between apical leaf symptoms and long-term root phenotypes for wildtype GHu infected plants.

### Reactive oxygen species are produced in roots during GFLV infection

**Fig. 4:**
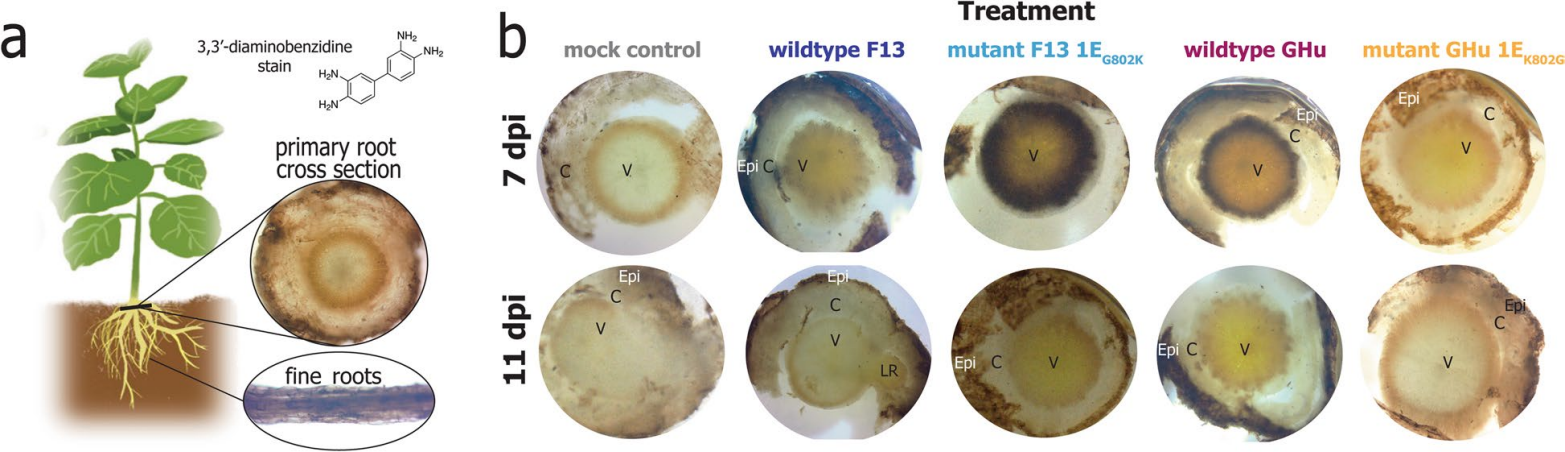
Reactive oxygen species (ROS) transiently produced in *Nicotiana benthamiana* primary root pericycle by four grapevine fanleaf virus (GFLV) strains (i.e., wildtypes GHu and F13, and mutants GHu 1E^Pol*/Sd^_K802G_ and F13 1E^Pol*/Sd^_G802K_) at 7- and 11- days post-inoculation (dpi). **a** Anatomical view of primary root cross sections from *N. benthamiana* immediately stained with 3′3-diaminobenzidine, dark portions represent tissue with high amounts of ROS reacting with the stain. Fine roots produced high amounts of ROS as expected from rapidly growing root tissue. **b** Primary root cross sections of virus treatments display high amounts of precipitated 3–3’-diaminobenzidine stain in the pericycle for both wildtype GHu and mutant F13 1E^Pol*/Sd^_G802K_ at 7 dpi and a diminished ROS abundance at 11 dpi. All other treatments have much lower levels of ROS detectable within the vasculature and cortex at both time points. (Epi = root epidermis, C = cortex, V = vascular bundle, LR = lateral root primordium). Images are representative of at least three individuals (n = 3) of five sacrificed plants per treatment

ROS production was monitored in root tissue of *N. benthamiana* via histological cross sections of primary roots followed by DAB staining at 7 dpi (*n* = 25) and 11 dpi (*n* = 25) (Fig. 4). Filamentous roots were very dark from DAB staining, likely due to the stress of digging up the plants from the soil and the abundance of ROS in emerging root tips (Fig. 4a). At 7 dpi, roots of plants infected with wildtype GHu and mutant F13 1E^Pol*/Sd^_G802K_ contained a ring of dark stain bordering the pericycle and vascular bundle, but not the cortex (Fig. 4b). This was not observed for any GFLV strain at 11 dpi. Roots of plants infected with wildtype F13 and mutant GHu 1E^Pol*/Sd^_K802G_ did not have the same level of contrast but showed stain slightly darker than roots of control plants in the outermost parts of the vascular bundle (Fig. 4b). Mock control plants at 7 dpi (n = 5) and 11 dpi (n = 5) yielded minimal background staining of any vasculature or cortex, but epidermal tissue consistently showed a dark brown or black color. These results suggested that the nature of residue 802 of protein 1E^Pol*/Sd^ largely influences the amount of ROS produced in the primary roots of *N. benthamiana.*

### 3’RNA-sequencing, and data curation

Twenty-seven *N. benthamiana* root samples were sequenced on Illumina 500/550 platform, three replicates of each treatment (wildtype GHu, mutant GHu 1E^Pol*/Sd^_K802G_, mock control) at three time points (4-, 17-, 26 dpi). The average number of total reads per sample was 7,338,802. After identification of reads passing quality control thresholds and proper adapter trimming, an average of 7,271,449 reads remained per sample. Alignment to the Niben v1.0.1 genome [34] resulted in an average of 74.63% reads per sample, after outlier removal of sample mutant GHu 1E^Pol*/Sd^_K802G_ 17 dpi replicate #3 (low alignment rate ~ 6%). Alignment to the Nbe_v1 genome resulted in an average of 5% less aligned reads, therefore, downstream analysis utilized the Niben v1.0.1 alignment. Quality control as reported through FastQC analysis of RNA-sequencing files returned average sequence lengths of 86 base pairs, 36–39% GC content, and Phred quality scores maintaining a majority above 30, with many sequences at 35 (1 in 5000 base pair call inaccuracy), which were all acceptable for downstream analysis (Supplementary Table 7). Re-annotation of the *N. benthamiana* genome was performed to retrieve 11,525 GO terms that matched to the Niben v1.0.1 protein models (Supplementary Fig. 1).

### Initial statistical considerations of the 3’RNA-Seq dataset

FeatureCounts files were parsed to produce a gene count matrix that was utilized with a metadata matrix containing treatment, time, and a group factor of treatment*time for DESeq2 input. The resulting matrix represented 37,784 genes with a threshold of at least 20 counts across all samples. Count filtering, dispersion of counts, hierarchal clustering, and principal component analysis were performed on data prior to removal of mutant GHu 1E^Pol*/Sd^_K802G_ replicate #3 at 17 dpi, because it showed large separation from all other samples (Supplementary Fig. 8a-d). After removal of this outlier, the analysis pipeline continued with the remaining 26 samples. Normalized counts returned roughly ~ 200,000 reads per sample across all time points and treatments (Supplementary Fig. 9a). Further, dispersion estimate curves were largely normal across the DESeq2 experiment, indicating that the normalization of counts and quality of reads were acceptable. A strong grouping by both treatment and time in hierarchal clustering and principal component analysis was observed (Supplementary Fig. 9c-d). All mock control samples grouped away from GFLV-infected samples, and GFLV-infected samples separated by time point through McQuitty method hierarchal clustering (Supplementary Fig. 9c). Further, samples were discriminated by treatment and time through principal component analysis with PC1 of 29.89% and PC2 of 12.42%. Samples at 4 dpi grouped in the middle of the plot, loosely associated, while samples at 17 dpi grouped to the right of the plot. Samples collected at 26 dpi were located on the left of the PCA plot in tight association, suggesting that these samples were differentiated from the other time points based on PC1, but less discriminated by PC2. Based on these results, the dispersion of counts being acceptable after normalization (Supplementary Fig. 9c), and the successful alignment of 201,108 reads per sample, data were deemed acceptable for further analysis.

### Differential transcript abundance analysis

**Fig. 5:**
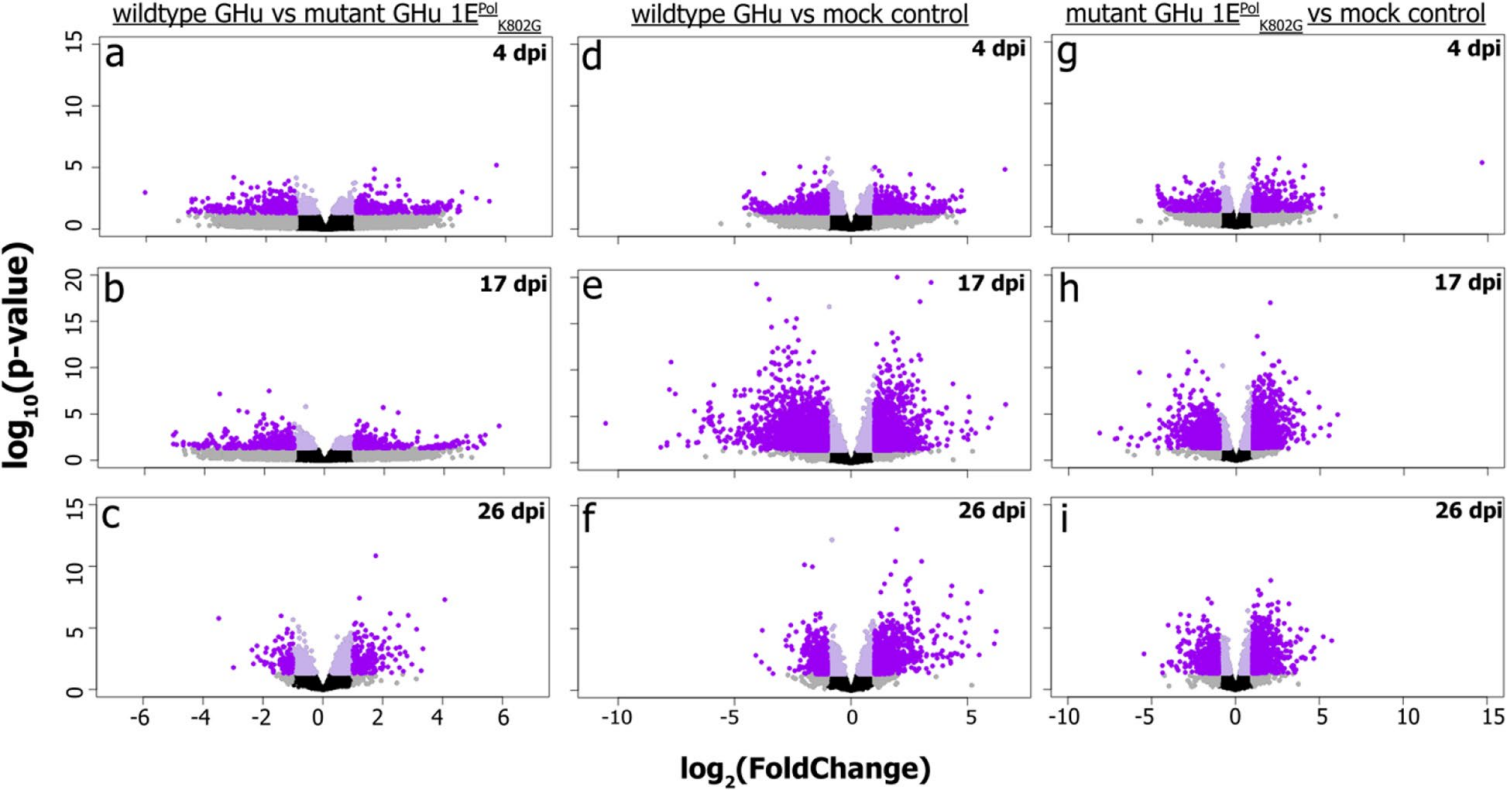
Volcano plot contrasts of *Nicotiana benthamiana* plants infected with two grapevine fanleaf virus (GFLV) strains (i.e., wildtypes GHu and mutant GHu 1E^Pol*/Sd^_K802G_) reveal perturbations of the root transcriptome at three time points; 4-, 17- and 26 days post-inoculation (dpi). Contrasts are divided by treatment in columns such as wildtype GHu vs mutant GHu 1E^Pol*/Sd^_K802G_ (**a**, **b**, **c**), wildtype GHu vs mock control (**d**, **e**, **f**), and mutant GHu 1E^Pol*/Sd^_K802G_ vs mock control (**g**, **h**, **i**) and by time (rows) at 4 dpi (**a**, **d**, **g**), 17 dpi (**b**, **e**, **h**) and 26 dpi (**c**, **f**, **i**). Logarithmic transformation of transcript abundance comparisons is shown on the x-axis. Confidence values as logarithmic transformations of *p*-value are represented on the y-axis. Genes shown as purple data points meet qualifications of *p*-value < 0.05 and absolute Log2FoldChange ≥ 1. Genes in gray meet only one qualification, while black data points denote neither qualification was met

Contrasts were generated within the DESeq2 package to examine differences in the transcriptome between treatments. Thresholds of *P* < 0.05 and |Log2FoldChange|> 1 were used for determining differentially abundant genes (DEGs). When comparing wildtype GHu to mutant GHu 1E^Pol*/Sd^_K802G_ at 4-, 17- and 26 dpi, a total of 704, 761, and 1041 transcripts was found to be differentially abundant, respectively (Table 1, Fig. 5a-c). An increased number of differentially abundant transcripts was obtained when comparing viral treatments to the mock controls at each time point. When comparing wildtype GHu to mock control transcripts, a total of 713, 4351, and 1692 transcripts were differentially abundant at 4-, 17- and 26 dpi, respectively (Table 1, Fig. 5 d-f). Lastly, when comparing mutant GHu 1E^Pol*/Sd^_K802G_ to mock control, a total of 437, 3293, and 2124 DEGs were found at 4-, 17-, and 26 dpi, respectively (Table 1, Fig. 5g-i).

**Table 1:** Differentially abundant transcripts as compared through DESeq2 analysis of *Nicotiana benthamiana* plant roots infected with two grapevine fanleaf virus strains, i.e., wildtype GHu and mutant GHu 1E^Pol*/Sd^_K802G_) at 4-, 7- and 11 days post-inoculation (dpi). DEGs were defined as having both a p-value < 0.05 and an absolute Log2FoldChange > 1

Treatment contrast	wildtype GHu vs mutant GHu 1E^Pol*/Sd^_K802G_		wildtype GHu vs mock control		mutant GHu 1E^Pol*/Sd^_K802G_ vs mock control	
Time	UP	DOWN	UP	DOWN	UP	DOWN
4 dpi	332	372	371	342	264	173
17 dpi	318	443	1548	2803	1483	1810
26 dpi	586	455	1043	649	1162	962

The top ten *N. benthamiana* root genes of differential abundance per treatment contrast and time point were identified (Table 2). By contrasting wildtype GHu and mutant GHu 1E^Pol*/Sd^_K802G_, host root genes relating to immune response, signaling, disease resistance, hydrolase activity, and components of viral silencing were consistently found to be dysregulated. Transcripts for regulators of transcription, translation, and post-translational processes were most abundant (Table 2). When contrasting wildtype GHu to mock inoculated plants, host genes relating to transcription, mitochondria and signaling patterns, protein synthesis, and immune response were dysregulated in the top 10 DEGs across all time points. Further, glutamine synthase and phosphatidylinositol-related genes appeared in the top 10 DEGs at 17 dpi, consistent with previously identified candidates in apical leaf tissue during GFLV infection and foliar symptom development [23]. Additional regulators of transcription and translation, immune response and signaling genes were dysregulated in the contrast of mutant GHu 1E^Pol*/Sd^_K802G_ and mock control across the three time points. Across all contrasts, genes relating to innate immune response were consistently present, suggesting residue 1E^Pol*/Sd^_802_ impacts the level in which immune responses are activated during infection. Notably, five unknown proteins were dysregulated to high magnitudes up to Log2FC of 14.66. These genes could have important functions related to morphology or viral infection not yet characterized in plant root tissue. All differentially abundant transcripts were documented (Supplementary Table 8).

**Table 2:** The top ten differentially abundant transcripts present in each viral contrast with their respective gene ID, name, Log2FoldChange, and *p*-value, ranked by p-value

Sheet 1. Wildtype GHu contrasted with mutant GHu 1E^Pol*/Sd^_K802G_ at 4-, 17-, and 26 days post inoculation in *Nicotiana benthamiana*						
Time	Rank	Name	GeneID	Log2FoldChange	lfcSE	*p*-value
4 dpi	1	Mitochondrial protein	Niben101Scf04451g00027	5.6966875	1.2629942	6.47E-06
	2	Protein NRT1/ PTR FAMILY 4.3	Niben101Scf04119g03004	1.6205678	0.3728127	1.38E-05
	3	Isoflavone reductase family protein	Niben101Scf04113g17021	-3.0801945	0.7699946	6.33E-05
	4	Mitochondrial substrate carrier family protein	Niben101Scf02168g04008	-0.9887381	0.2482862	6.83E-05
	5	LOB domain-containing protein 41	Niben101Ctg14421g00002	1.6113985	0.4074446	7.66E-05
	6	Protein translocase subunit SecA	Niben101Scf01888g03004	2.4184992	0.6197774	9.53E-05
	7	Glutamate receptor 2.8	Niben101Scf00747g12032	-1.250575	0.3255952	1.23E-04
	8	FAD-binding Berberine family protein	Niben101Scf09217g00016	0.9738886	0.2592972	1.73E-04
	9	Proteinase inhibitor type-2	Niben101Scf07757g00001	-2.8053858	0.7484557	1.78E-04
	10	ATP-dependent RNA helicase eIF4A	Niben101Scf01998g00010	-1.8775588	0.5017108	1.82E-04
Time	Rank	Name	GeneID	Log2FoldChange	lfcSE	*p*-value
17 dpi	1	Protein kinase superfamily protein	Niben101Scf01450g01008	-1.8437026	0.3341768	3.45E-08
	2	Disease resistance protein I2	Niben101Scf03396g07026	-3.4940569	0.6487701	7.22E-08
	3	ABC transporter ATP-binding protein ARB1	Niben101Scf15836g04006	-0.6199358	0.129438	1.67E-06
	4	Beta-amylase	Niben101Scf10158g01036	1.9664738	0.4140633	2.04E-06
	5	Cytochrome c oxidase subunit 2	Niben101Scf04412g00017	-2.8547363	0.6198686	4.12E-06
	6	Xyloglucan endotransglucosylase/hydrolase protein 22	Niben101Scf14470g00001	-2.5754905	0.5690913	6.02E-06
	7	Patatin-like protein 7	Niben101Ctg15717g00003	2.465677	0.5483163	6.90E-06
	8	Unknown protein	Niben101Scf06105g01010	-2.0285676	0.4610409	1.08E-05
	9	40S ribosomal protein S4	Niben101Scf17473g00009	-1.4071685	0.3344886	2.59E-05
	10	glucan synthase-like 4	Niben101Scf00790g00004	-2.016389	0.4822456	2.90E-05
Time	Rank	Name	GeneID	Log2FoldChange	lfcSE	*p*-value
26 dpi	1	Glutamine synthetase	Niben101Scf00345g01021	1.7674997	0.2615045	1.39E-11
	2	Serine carboxypeptidase II-2	Niben101Scf02359g00009	1.2220252	0.2221445	3.78E-08
	3	Cupredoxin superfamily protein	Niben101Scf02393g02025	4.0739648	0.7475112	5.04E-08
	4	Pyrimidine-specific ribonucleoside hydrolase RihA	Niben101Scf04674g02018	2.2507337	0.4528093	6.67E-07
	5	nodulin MtN21 /EamA-like transporter family protein	Niben101Scf09197g00005	2.8514417	0.581598	9.45E-07
	6	Actin-2	Niben101Scf00672g00001	-1.392489	0.2851484	1.04E-06
	7	Pectinesterase 3	Niben101Scf03706g02006	-3.4769013	0.725795	1.66E-06
	8	Double-stranded RNA-binding protein 2	Niben101Scf01759g04003	-0.9927502	0.209516	2.16E-06
	9	Apyrase	Niben101Scf05918g03001	1.1004463	0.2379406	3.75E-06
	10	FAS-associated factor 2-B	Niben101Scf02972g07001	1.054296	0.231198	5.11E-06
Sheet 2. Wildtype GHu contrasted with mock inoculated controls at 4-, 17-, and 26 days post inoculation in *Nicotiana benthamiana*.						
Time	Rank	Name	GeneID	Log2FoldChange	lfcSE	*p*-value
4 dpi	1	Unknown protein (BLAST returns Flocculation protein)	Niben101Scf03494g00002	16.8271419	3.1119877	6.40E-08
	2	Heat shock 70 kDa protein 1A	Niben101Scf05389g00010	-0.992725	0.2053522	1.34E-06
	3	Mitochondrial protein	Niben101Scf04451g00027	6.6042454	1.3974789	2.29E-06
	4	Mitochondrial substrate carrier family protein	Niben101Scf02168g04008	-1.1086204	0.2481061	7.88E-06
	5	DNA-binding protein [Streptococcus suis]	Niben101Scf03528g03009	1.0295563	0.230432	7.90E-06
	6	ATP-dependent RNA helicase eIF4A	Niben101Scf01998g00010	-2.1936224	0.4975499	1.04E-05
	7	Thermosome subunit	Niben101Scf13415g00021	0.9343139	0.2119629	1.04E-05
	8	50S ribosomal protein L11	Niben101Scf01911g02007	-0.8416185	0.1963029	1.81E-05
	9	Heat shock 70 kDa protein 1-like	Niben101Scf12154g01009	-1.1372504	0.2653934	1.83E-05
	10	Tudor/PWWP/MBT superfamily protein	Niben101Scf01722g04010	1.2035777	0.2816975	1.93E-05
Time	Rank	Name	GeneID	Log2FoldChange	lfcSE	*p*-value
17 dpi	1	Sec14p-like phosphatidylinositol transfer family protein	Niben101Scf01018g03001	1.9836672	0.2093088	2.61E-21
	2	GATA transcription factor 2	Niben101Scf08635g02001	3.4384291	0.3744416	4.20E-20
	3	Low PSII Accumulation 3 isoform 3	Niben101Scf00715g09005	-4.0551673	0.4448789	7.85E-20
	4	cAMP-regulated phosphoprotein 19-related protein	Niben101Scf22015g02047	-0.9337399	0.1056044	9.41E-19
	5	Pentatricopeptide repeat-containing protein	Niben101Scf02525g02002	-3.5162577	0.4035648	2.96E-18
	6	UDP-D-glucuronate 4-epimerase 6	Niben101Scf02104g01010	2.9642438	0.3402434	2.98E-18
	7	evolutionarily conserved C-terminal region 5	Niben101Scf08176g00008	-2.337806	0.2850484	2.38E-16
	8	glucan synthase-like 4	Niben101Scf02725g02025	-2.7706434	0.3427949	6.34E-16
	9	Mitochondrial substrate carrier family protein	Niben101Scf02268g05007	-2.4230657	0.3058151	2.31E-15
	10	glucan synthase-like 4	Niben101Scf00790g00004	-3.4114818	0.430985	2.46E-15
Time	Rank	Name	GeneID	Log2FoldChange	lfcSE	*p*-value
26 dpi	1	Glutamine synthetase	Niben101Scf00345g01021	1.9631991	0.2618972	6.58E-14
	2	cAMP-regulated phosphoprotein 19-related protein	Niben101Scf22015g02047	-0.8186601	0.1099428	9.60E-14
	3	Glutamine synthetase	Niben101Scf01765g02012	1.9044151	0.2854461	2.53E-11
	4	Isoaspartyl peptidase/L-asparaginase	Niben101Scf05643g05001	3.032	0.4587894	3.88E-11
	5	expressed protein [Oryza sativa Japonica Group]	Niben101Scf04259g01024	-1.9964343	0.3061783	7.01E-11
	6	myb-like transcription factor family protein	Niben101Scf03942g02002	-1.6564299	0.2544056	7.47E-11
	7	5-methyltetrahydropteroyltriglutamate--homocysteine methyltransferase	Niben101Scf09725g00022	1.7118145	0.2710982	2.71E-10
	8	Major facilitator superfamily protein	Niben101Scf04223g00001	2.5284428	0.4114759	8.01E-10
	9	Phospholipase A1-II 1	Niben101Scf10353g00011	2.4200058	0.3973735	1.13E-09
	10	Apyrase	Niben101Scf05918g03001	1.4397588	0.2381754	1.49E-09
Sheet 3. Mutant GHu 1E^Pol^^*/Sd^_K802G_ contrasted with mock inoculated controls at 4-, 17-, and 26 days post inoculation in *Nicotiana benthamiana*.						
Time	Rank	Name	GeneID	Log2FoldChange	lfcSE	*p*-value
4 dpi	1	Unknown protein	Niben101Scf00239g04001	1.3152369	0.2833667	3.46E-06
	2	Protein trichome birefringence-like 19	Niben101Scf02215g01004	2.5423049	0.5559715	4.81E-06
	3	Unknown protein	Niben101Scf03494g00002	14.6555401	3.2427267	6.20E-06
	4	Adenylyl cyclase-associated protein	Niben101Scf13199g00006	-0.8376836	0.1854987	6.31E-06
	5	purple acid phosphatase 27	Niben101Scf03184g02002	1.293926	0.288435	7.26E-06
	6	Heat shock 70 kDa protein 1A	Niben101Scf05389g00010	-0.9033857	0.2042308	9.72E-06
	7	Unknown protein	Niben101Scf01881g02004	1.0296128	0.2370674	1.40E-05
	8	Proteinase inhibitor I-A	Niben101Scf09044g01003	4.0785915	0.9449667	1.59E-05
	9	Beta-D-xylosidase 4	Niben101Scf01894g02004	1.1060834	0.2607789	2.22E-05
	10	Leucine-rich repeat (LRR) family protein	Niben101Scf02422g02015	-0.8890076	0.2130283	3.00E-05
Time	Rank	Name	GeneID	Log2FoldChange	lfcSE	*p*-value
17 dpi	1	Sec14p-like phosphatidylinositol transfer family protein	Niben101Scf01018g03001	2.0325732	0.2335843	3.27E-18
	2	Calcium-binding protein 2	Niben101Scf01002g02005	1.251441	0.1616167	9.69E-15
	3	Heat shock 70 kDa protein 1A	Niben101Scf05389g00010	1.622938	0.2297735	1.63E-12
	4	Unknown protein	Niben101Scf00321g02008	-2.8658029	0.408885	2.40E-12
	5	cAMP-regulated phosphoprotein 19-related protein	Niben101Scf22015g02047	-0.8036557	0.1185462	1.21E-11
	6	Myb-related protein 306	Niben101Scf01013g01003	-2.4164981	0.3585196	1.58E-11
	7	UDP-D-glucuronate 4-epimerase 6	Niben101Scf02104g01010	2.5510918	0.3794764	1.78E-11
	8	Nutrient reservoir [Cucumis sativus]	Niben101Scf16997g00001	2.2587796	0.3442533	5.33E-11
	9	Beta-D-xylosidase 4	Niben101Scf01894g02004	2.1428993	0.3275848	6.09E-11
	10	Dormancy/auxin associated family protein	Niben101Scf01041g04001	-2.8642541	0.4492301	1.82E-10
Time	Rank	Name	GeneID	Log2FoldChange	lfcSE	*p*-value
26 dpi	1	WRKY transcription factor 1	Niben101Scf01833g00002	2.1014269	0.346101	1.27E-09
	2	Glycerol-3-phosphate dehydrogenase [NAD(P)+]	Niben101Scf01612g02014	1.3518906	0.2329634	6.51E-09
	3	Cyclin-U2-2	Niben101Scf00397g06001	1.5115199	0.2660277	1.33E-08
	4	Unknown protein	Niben101Scf01220g04008	1.4435006	0.2555505	1.62E-08
	5	Unknown protein	Niben101Scf04259g01024	-1.6340773	0.2988318	4.55E-08
	6	Boron transporter 1	Niben101Scf06412g01004	-1.4374495	0.2680706	8.22E-08
	7	Pentatricopeptide repeat-containing protein	Niben101Scf03076g02006	2.5436469	0.477935	1.03E-07
	8	Neutral ceramidase	Niben101Scf00978g09002	1.1002245	0.2070326	1.07E-07
	9	Ubiquitin-fold modifier-conjugating enzyme 1	Niben101Scf02593g00002	1.314139	0.2483603	1.21E-07
	10	Organ-specific protein P4	Niben101Scf03839g12008	3.1856642	0.6040954	1.34E-07

### Gene set enrichment analysis

From each of the treatment contrasts, DEGs were extracted and subject to GSEA to detect overrepresented gene ontology terms. An ordered list of DEGs was used to produce enriched terms between each contrast at each respective time point (Supplementary Fig. 10). Few terms were produced during contrasts of wildtype GHu and mutant GHu 1E^Pol*/Sd^_K802G_ root transcriptome profiles to include response to external stimulus (GO:0009605) and pigment binding (GO:0031409) at 4 dpi, negative regulation of plant-type hypersensitive response (GO:0034051) at 17 dpi, and regulation of steroid metabolic process (GO:0019218) at 26 dpi. The relatively low number of DEGs detected between these two treatment contrasts might have prevented further resolution of these data related to the observed changes in RSA.

**Fig. 6:**
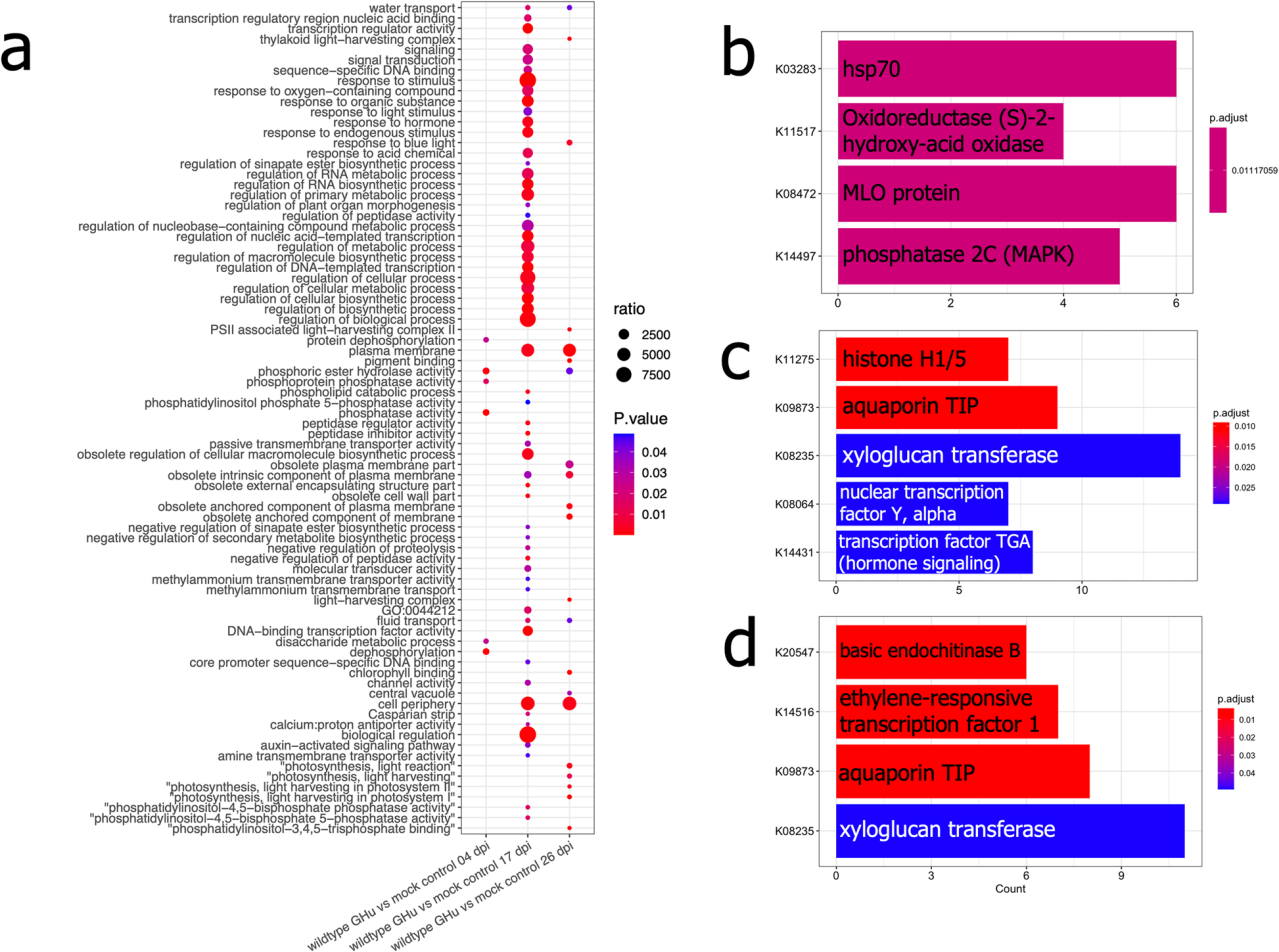
Downstream overabundance of transcript dysregulation in *Nicotiana benthamiana* roots infected with wildtype grapevine fanleaf virus strain GHu through gene set enrichment analysis and Kyoto Encyclopedia of Genes and Genomes (KEGG) reveals several conserved pathways of overabundance. **a** The dotplot representation of wildtype GHu infected plants against mock control GSEA shows pathways dysregulated at all three time points past inoculation. At early infection, 4 days post inoculation (dpi), categories related to signaling and phosphorylation are displayed. At 17 dpi, many components of metabolism, immune pathways, and signaling are overrepresented. Finally at 26 dpi, components related to physiology, photosynthesis, and signaling were significantly overrepresented. The size of each data point represents the ratio of genes dysregulated in this category related to the total number of genes in the genome. **b** KEGG analysis shows similar pathways at early infection for specific signaling and innate immune encoding genes. **c** At 17 dpi unique signaling molecules through gene regulation were overrepresented in addition to structural and transport genes. **d** Finally, similar genes were found at 26 dpi to 17 dpi, with the addition of differential abundance of plant hormone pathways and a general response factor chitinase

Many more gene ontologies were detected through GSEA by contrasting wildtype GHu with mock inoculated control plants. At 4 dpi, ontologies related to dephosphorylation (GO:0016311) and phosphatase activity (GO:0016791) amongst other closely related GOs were overrepresented. Out of 59 total terms at 17 dpi, the top three GO’s overrepresented according to p-value were DNA-binding transcription factor (GO:0003700), transcription regulator activity (GO:0140110), and response to stimulus (GO:0050896). Several terms relating to biosynthetic processes, phosphorylation, response to oxygen species, and hormone response were overrepresented at this time point as well (Fig. 6a). Pigment binding (GO:0031409), obsolete anchored component of membrane (GO:0031225), and photosynthesis, light harvesting in photosystem I (GO:0009768) were the top three overrepresented GOs at 26 dpi (Fig. 6a). Further terms related to photosynthetic components and secondary metabolite regulation were found at 26 dpi.

Contrasting mutant GHu 1E^Pol*/Sd^_K802G_ and mock inoculated control plants revealed gene ontologies that were reminiscent of viral infection without all changes occurring to root morphology, as observed through the previously described RSA phenotyping (Supplementary Fig. 10). Thylakoid (GO:0009579), photosynthetic membrane (GO:0034357), and singlet oxygen-mediated programmed cell death (GO:0010343) were among the overrepresented, non-redundant GO’s present at 4 dpi. At 17 dpi, plasma membrane (GO:0005886), cell periphery (GO:0071944), and response to stimulus (GO:0050896) were the top three overrepresented GOs by p-value, with other terms relating to hormone response and phosphorylation activity also being abundant at this time. At 26 dpi, 29 terms were overrepresented with the top three by p-value being regulation of hormone levels (GO:0010817), hormone metabolic process (GO:0042445), and lipid metabolic process (GO:0006629). Other closely related terms of response to stimulus, plasma membrane, and phosphorylation were also present during late infection (Supplementary Fig. 10).

A secondary evaluation of enrichment for differentially abundant transcripts found in roots was performed through Kyoto Encyclopedia of Genes and Genomes (KEGG) analysis. Significant KEGG terms were defined by the thresholds of *p* < 0.05 and *q* < 0.05 to evaluate the 3’RNA-Seq differentially abundant genes against the newly curated genome annotation. The enriched KEGG terms confirmed previous GSEA results to display terms of immune response, gene regulation, and signal transduction (Fig. 6b-d, Supplementary Fig. 11). Signaling genes such as calcium-binding protein CML (ko:k13448), phosphatase 2C of the MAP kinase pathway (ko:k14497), and calcium dependent protein kinase (ko:k13412) were present at various contrasts and time points during GFLV infection (Supplementary Fig. 11a,d,h). Interestingly, a new term relating to cell elongation and division appeared as well throughout multiple contrasts and time points: xyloglucan transferase (ko:k08235) (Supplementary Fig. 11b,e,f). The results of the KEGG analysis further documented phytohormone pathways (ethylene, abscisic acid, transcription factor TGA) to be impacted upon viral infection although their relation to the development of root symptoms may not be immediately apparent. Several terms related to protein repair such as chaperone proteins, oxidases, and hormone regulators were found at 26 dpi from KEGG analysis (Supplementary Fig. 11c,f,i).

### Dysregulation of host pathways associated with viral infection and root symptom development

**Fig. 7:**
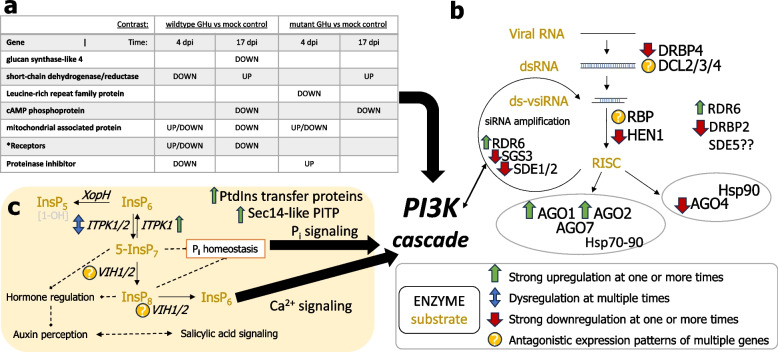
The dysregulation of three major pathways in the roots of *Nicotiana benthamiana* infected by grapevine fanleaf virus (GFLV) revealed by ontology and enrichment analysis. **a** Immune receptors and innate immune pathways were disrupted at 4- and 17-days post inoculation (dpi) when comparing viral treatments to mock inoculated plants. Further, wildtype GHu infected plants were more dysregulated across multiple types of innate immune pathways that feed into the PI3K cascade. **b** The host viral silencing pathway had several genes dysregulated throughout the course of GFLV infection, in that, either both viral treatments dysregulated or wildtype GHu altered expression to a greater extent. **c** Inositol biosynthesis was dysregulated in collected root RNA samples throughout the course of infection, in which the downstream effects of inositol metabolites can have profound signaling capabilities in hormone regulation, inorganic phosphate signaling, and calcium signaling that can amplify the PI3K cascade. Ultimately, these three pathways converge on the hallmarks of hypersensitive response during plant-pathogen interactions

The three major host pathways resolved from GSEA, KEGG, and top candidates of differential abundance analysis were: (i) regulation of gene expression, (ii) immune signaling and oxidative response to pathogen invasion, and (iii) production of secondary metabolites (Fig. 6). Substantial overlap of these pathways was previously identified in apical leaf tissue through transcriptomic and proteomic analysis [23]. A fourth pathway of interest was considered based on the recent description of GFLV suppression of RNA silencing (14). From top gene candidates in root RNA-Seq analysis, immune signaling pathways were highly overrepresented, especially innate immune receptors (positive and negative dysregulation), cAMP phosphoprotein downregulated in both viral contrasts at 17 dpi, and proteinase inhibitors negatively regulated for wildtype GHu while upregulated for mutant GHu 1E^Pol*/Sd^_G802K_ when compared against the mock controls (Fig. 7). Several host-encoded gene silencing transcripts, including suppressor of gene silencing 3, SDE 1 and 2, AGO4, HEN1, and DRBP4, were less abundant in GFLV-infected *N. benthamiana* roots. Other members of the RNA-induced silencing complex such as AGO1 and AGO2 were upregulated, but other transcripts were either too low in number or provided orthologs with conflicting patterns to deduce a trend (Fig. 7).

## Discussion

In this study, we documented changes in RSA traits in *N. benthamiana* upon GFLV infection (Figs. 1 and 2). The average number of *N. benthamiana* root tips decreased by up to a third and the average root diameter increased over 20% upon infection by wildtype GHu and mutant F13 1E^Pol*/Sd^_G802K_ when compared with roots of mock inoculated plants (Fig. 1a,b). The RNA1 of these two GFLV strains differ by 17% at the nucleotide sequence level [20]; however, the RNA1-encoded protein 1E^Pol*/Sd^ of both wildtype GHu and mutant F13 1E^Pol*/Sd^_G802K_ carry a lysine in position 802 [21]. In contrast, *N. benthamiana* plants infected with wildtype F13 and mutant GHu 1E^Pol*/Sd^_K802G_, both carrying a glycine at position 802 of protein 1E^Pol*/Sd^ [21], displayed RSA traits resembling those of mock control plants. These findings indicate that a single GFLV amino acid is associated with changes in RSA traits rather than other genetic differences of up to 17% between wildtype F13 and GHu. This is the first report of a single amino acid of a secovirus altering RSA traits of a plant host.

Efforts to correlate the vein clearing phenotype in apical leaves upon infection of *N. benthamiana* with wildtype GHu to RSA traits showed that the duration of foliar symptoms was significantly positively correlated with root diameter traits, and significantly negatively correlated with branching patterns, root tip number, and network metrics (Fig. 3a, Supplementary Fig. 3). Linear models confirmed these correlated above and below ground symptoms (Fig. 3d,e). The other three strains of GFLV used in this study were asymptomatic in *N. benthamiana* leaves [20, 21, 23], preventing any comparison between above and below ground features.

The root phenotype was correlated to ROS produced in the pericycle of the primary roots (Fig. 4) and the dysregulation of the host transcriptome (Fig. 5). Abundant ROS production was specific to root systems infected with GFLV strains containing K at residue 1E^Pol*/Sd^_802_ (Fig. 4) with transcriptomic analysis confirming an active regulation of these transcripts across viral treatments (Fig. 6). There was a direct correlation between the number of days showing symptoms in apical leaves and the severity of changes in RSA traits for plants infected by wildtype GHu (Fig. 3), while a correlation of ROS production was shown in the roots infected with wildtype GHu and mutant F13 1E^Pol*/Sd^_G802K_ (Fig. 4B). These results suggested a conserved reaction across tissue types that might be required for GFLV systemic infection and perhaps an evolutionarily conserved mechanism that elicits symptoms in *N. benthamiana*. Previous studies found that additional modifications to the amino acids in this region often reduced pathogenicity or virulence, or completely abolished the infectivity of the virus, indicating this protein’s critical involvement in the infection process [21, 52].

While several new host gene candidates were identified as most differentially abundant in root tissue of GFLV-infected *N. benthamiana*, general trends remained like those previously identified in apical leaves [23]. The enrichment term of singlet oxygen-mediated programmed cell death (GO:0010343) was present in all GFLV-infected plants tested in this study. This result further supports the hypothesis that all GFLV strains elicit a certain level of ROS with symptomatic strains being the most active. Additionally, five genes specified as PROTEIN OF UNKNOWN FUNCTION were among the top differentially abundant transcripts. Many of these candidate genes could benefit from follow up functional validation analysis in knock-out assays, with confirmation of gene classification and identification if resultant functions are specific to root tissue. The continued functional validation of root specific transcripts will aid in systems biology analyses for plant-pathogen interactions and provide genetic improvement resources for plant biologists [53].

Curated transcriptomics data and our updated annotation informed molecular mechanisms of the resultant *N. benthamiana* root phenotypes upon GFLV infection (Fig. 7). In roots, upregulation of inositol-tetrakisphophate 1/2 kinase and the antagonistic expression patterns of other inositol synthesis genes prevented the full realization of a mechanism, although an imbalance of this signaling molecule could explain similar events occurring for the transient ROS production in root tissue (Fig. 7). Rather than a 1:1 model, we hypothesize synergistic effects of inositol-mediated signaling, immune receptors, and hormones to contribute to the observed root modifications. Interestingly, the top DEGs return to phosphatidylinositol and extended pathways that relate to the PI3 kinase cascade. This central innate immune response regulator is widely involved in host–pathogen interactions. The top gene according to p-value at 17 dpi when contrasting wildtype GFLV-GHu to mock control is the SEC14P-LIKE PHOSPATIDYLINOSITOL TRANSFER FAMILY PROTEIN, with a Log2FoldChange of 1.98 (Table 2). This gene is a key regulator of many downstream biological processes [54], further supporting the proposed hypersensitive response during GFLV infection. As with the innate mammalian immune system [55], the PI3K cascade was identified as a major factor in bacterial plant-pathogen hypersensitive response [56]. It is notable that genes related to this pathway were documented in both shoot [23] and root tissue (this study), making evident that the combination of signaling pathways are utilized to mount the robust production of ROS (Fig. 7). Future work could address the benefit of ROS accumulation to the host and the pathogen, and how this cascade might have influenced the evolutionary adaptation of the virus in plant hosts.

It is known that plant viruses proficiently manipulate host gene regulatory pathways [57]. Generally, transcriptional and translational regulators can be activated or repressed, directly or indirectly, by RNA/protein binding plant hormones, metabolites, or proteins in plant root tissue. Some factors described in the literature to great extents include abscisic acid, indole 3-butyric acid, ethylene, gibberellic acid, brassinosteroids, nitrogen, glutamate, nitrous oxide, phosphate, sulfate, temperature, and water availability [58–60]. The manipulation of one or more of these factors can alter the way in which root systems are formed, usually in a dose-dependent manner. The underlying molecular mechanisms behind abiotic factor changes have been identified in many systems from model plants like *Arabidopsis thaliana* [61, 62] to perennial crop systems like grape, apple, and orange [60]. A summary of historically understood models expands upon gene regulation, hormone pathways, and nutrient availability to determine root structure [59]. The documentation of modified root morphology upon virus infection overlaps considerably with nutrient deficiencies/toxicities such as phosphorus [63], nitrogen [64], calcium [65], and others. As our study was performed in controlled conditions, the impacts of nutrient, light, or temperature differences were limited. Thus, root traits could be ascribed to a single amino acid of the GFLV-encoded protein 1E^Pol*/Sd^. We further hypothesize that the severity of symptoms occurs in a similar manifestation to dose-dependent concentrations of stressors (i.e., secondary signaling molecules, nutrient availability, plant hormones, etc.,), but in this instance to be dependent upon the protein encoding factor(s) of the virus exposed to the host machinery.

GFLV-host interactions documented at the root level by 3’RNA-Seq included multiple overlapping pathways of immune response and hormone regulation. The upregulation of oxidoreductases in roots at 17 dpi indicate lessened ROS levels and a diminished effect on plant response thereafter (Fig. 6). Tolerance of oxidative stress and respective signaling patterns to auxin mediate large morphological changes in plants [66], which might explain the root phenotype in GFLV-infected *N. benthamiana*. Overall, ROS production due to a hypersensitive response (HR) agrees with current understandings of virus-host interactions at the molecular level [67]. The regulation of HR is not linear and can utilize any combination of RNA silencing, pattern triggered immunity, and effector triggered immunity to elicit such responses [67]. Overall, HR, ROS burst, hormone manipulation are documented as common denominators in symptomatic viral infections [68] but the appearance and disappearance of ROS and symptomology above and below ground depending on a single viral amino acid is unique to GFLV.

Other plant systems have documented changes in RSA upon viral infection. For example, single infections of sweet potato chlorotic stunt virus or multiple infections of sweet potato feathery mottle virus, sweet potato virus 2, sweet potato virus C, and sweet potato virus G decrease lateral root development and adventitious roots of sweet potatoes (*Ipomoea batatas*) [2]. In a similar study, viral co-infections reduced the total marketable yield between 60–95% depending on the level of tolerance or susceptibility of the sweet potato variety [69]. Additionally, tomato (*Solanum lycopersicum* L.) infected with tomato brown rugose fruit virus differ in multiple RSA traits when compared with uninfected plants with a 15% reduction in root diameter, and a 30% decrease in root tip number at three weeks post inoculation [3]. Cassava brown streak virus (CBSV) causes root necrosis and tuber yield loss [70]. Almost all (99%) of above ground symptomatic cassava plants display necrotic lesions below ground due to the presence of CBSV, while only 16% of asymptomatic infection events above ground also have root necrosis [71]. Cassava mosaic disease is associated with at least ten different viral species and has been shown to have anywhere from minor changes to up to 87% losses in tuber yield in the most aggressive instances [72]. While cassava and sweet potato are tuber crops, significant impacts to above ground crop production can be associated with total root health, and thereby increasing the importance on documenting the most resilient and sturdy roots for prolonged cropping systems such as with perennial crops. In the case of BNYVV, three viral amino acids are crucial to determine a resistant or a susceptible phenotype in beets, with amino acid 68 of p25 being of critical stature for host-genotype interactions [73].

Our findings on GFLV strain-specific alterations in *N. benthamiana* RSA have direct implications in transmission biology. The ectoparasitic dagger nematode vector of GFLV, *X. index*, acquires GFLV in a non-circulative, non-propagative manner by feeding on infected plant roots [74–76]. We hypothesize that the phenotype of RSA induced by wildtype GHu and mutant F13 1E^Pol*/Sd^_G802K_ deter continued feeding of *X. index* on *N. benthamiana* roots. This is because plants with significantly less root tips and increased root diameter are less appetent to *X. index*, which prefers fibrous over mature roots for sustained feeding and reproduction [75]. Modified RSA traits of plants infected with wildtype GHu or mutant F13 1E^Pol*/Sd^_G802K_ could push nematode vectors away from an already infected host and increase the likelihood of feeding on neighboring healthy plants at a faster rate than unperturbed root systems of healthy plants or plants infected with other GFLV strains such as wildtype F13 or mutant GHu 1E^Pol*/Sd^_K802G_. Virus manipulation of plants and vectors to enhance acquisition and/or promote transmission to new plants is well documented for hemipteran-transmitted viruses [77]. It would be interesting to test how modified *N. benthamiana* RSA traits influence *X. index*-mediated GFLV transmission in controlled assays with different virus strains*.* Additionally, if modified RSA traits induced by specific GFLV strains are observed in the natural host *Vitis* spp., and *X. index* feeding strategies altered by GFLV-induced changes in root morphology are confirmed, new management strategies of GFLV based on changes in the host rhizosphere could be explored to reduce transmission events.

It is unknown if the GFLV-mediated root phenotype in *N. benthamiana* fully manifests in the natural host, *Vitis* spp. In the instance of BNYVV, the transient expression of protein p25 induced similar effects in *A. thaliana* as observed for the natural host, sugar beet [1, 73]. It would be interesting to investigate if distinct GFLV strains, including some that cause severe foliar symptoms in *Vitis* spp., affect RSA traits of infected grapevines over the vineyard’s lifespan. Similarly, a correlation analysis of above and below-ground traits in grapevines would be beneficial to better understand the mechanism of disease induced by GFLV. Such work could inform new management strategies for growers experiencing the impacts of fanleaf degeneration.

## Conclusions

We documented for the first time the impact of GFLV on RSA traits of infected *N. benthamiana*. The most notable changes in roots were observed in plants infected with wildtype GHu and mutant F13 1E^Pol*/Sd^_G802K_, both carrying a lysine at the 802nd position of the putative RNA-dependent RNA polymerase (protein 1E^Pol*/Sd^). These two GFLV strains caused a significant reduction in the number of root tips (up to 30%), an increased root average diameter (up to a 20%), and an abundant accumulation of ROS in the pericycle of the primary roots when compared with roots of mock inoculated control plants. The severity of root phenotypic changes was associated with ROS production in the root pericycle. Transcriptomic analysis revealed potential host gene candidates related to viral immune response, RNA silencing, and plant hormone pathways that could contribute to the observed root phenotype. More work is needed to understand any potential downstream impacts of root morphology, particularly on GFLV transmission by the ectoparasitic nematode vector *X. index*. Finally, unknown root transcripts dysregulated due to a single GFLV amino acid should be validated through reverse genetic assays to improve our understanding of how GFLV manipulates root architecture.

### Supplementary Information

Supplementary Material 1.

Supplementary Material 2.

Supplementary Material 3.

Supplementary Material 4.

Supplementary Material 5.

## Data Availability

All code and data for statistical analysis is provided as Supplementary File 1, as well a GitHub repository (https://github.com/brandon-roy/Root). Fastq files were deposited to NCBI under SRA accession PRJNA984296 (https://www.ncbi.nlm.nih.gov/sra/PRJNA984296). Any additional data or clarification can be requested of the corresponding author at any point, within reasonable request.
