# Supplementary material for: A single viral amino acid shapes the root system architecture of a plant host upon virus infection
Source: BMC Microbiol. 2024 Jul 19;24:267. doi: 10.1186/s12866-024-03399-x (PMC11264730; doi:10.1186/s12866-024-03399-x)
Supplement: Supplementary file 1 — Supplementary Material 1. [file 12866_2024_3399_MOESM1_ESM.docx]

**Supplementary documentation for:**

**A single viral amino acid shapes the root system architecture of a plant host upon virus infection**

Brandon G. Roy^1^† and Marc Fuchs^1^

^1^Cornell University, Plant Pathology and Plant-Microbe Biology Section, Cornell AgriTech at the New York State Agricultural Experiment Station, Geneva, NY 14456, USA.

†Corresponding author: [bgr36@cornell.edu](mailto:bgr36@cornell.edu)

**Data availability statement:**

All code and data for statistical analysis is provided as Supplementary File 1, as well a GitHub repository (<https://github.com/brandon-roy/Root>). Fastq files were deposited to NCBI under SRA accession PRJNA984296 (<https://www.ncbi.nlm.nih.gov/sra/PRJNA984296>). Any additional data or clarification can be requested of the corresponding author at any point, within reasonable request.

**Github page contains:**

**R Markdown Files (X)**

- Root phenotype quantification (4)
  - 4 dpi, 17 dpi (2), 26 dpi
- RSA combined and full trait visualization
- RNA-Seq database curation
- RNA-Seq virus detection
- RNA-Seq differential abundance analysis
- RNA-Seq functional enrichment analysis

**Dependency files formatted for use with each .Rmd file.**

**2 sets of FeatureCounts output**

**4 excel files with Rhizovision output**

**Supplementary Table 1.** Grapevine fanleaf virus (GFLV) RNA1 sequence analyses of DNA amplicons obtained from root tissue of *Nicotiana benthamiana* by RT-qPCR. Sequences were aligned with UGENE (v45.1) and consensus sequences were aligned to sequences of wildtype GFLV strains F13 and GHu. DPI = days post inoculation. Average alignment was 98.6% identity at the nucleotide level to sequences available in NCBI.

| **GFLV­ inoculum source** | **DPI** | **Primer** | **Reference** | **E-value** | **% Identity** | **Nt** | **802 ID**^a^ |
| --- | --- | --- | --- | --- | --- | --- | --- |
| mutant F13 1E^Pol^_G802K_ | 17 | FWD | NC_003615.1  (F13 RNA1) | 4.00E-99 | 98.98 | 395 | K |
| mutant F13 1E^Pol^_G802K_ | 17 | REV |  | 3.00E-100 | 99.49 | 414 | K |
| wildtype F13 | 17 | FWD |  | 2.00E-97 | 98.48 | 396 | G |
| wildtype F13 | 17 | REV |  | 8.00E-101 | 99.49 | 410 | G |
| mutant GHu 1E^Pol^_K802G_ | 17 | FWD | JN391442.1  (GHu RNA1) | 1.00E-94 | 97.96 | 205 | G |
| mutant GHu 1E^Pol^_K802G_ | 17 | REV |  | 1.00E-94 | 97.96 | 203 | G |
| wildtype GHu | 17 | FWD |  | 2.00E-97 | 98.01 | 202 | K |
| wildtype GHu | 17 | REV |  | 5.00E-98 | 98.97 | 204 | K |
| mutant F13 1E^Pol^_G802K_ | 26 | FWD | NC_003615.1  (F13 RNA1) | 1.00E-99 | 98.51 | 474 | K |
| mutant F13 1E^Pol^_G802K_ | 26 | REV |  | 1.00E-99 | 99.49 | 339 | K |
| wildtype F13 | 26 | FWD |  | 2.00E-97 | 98.48 | 394 | G |
| wildtype F13 | 26 | REV |  | 2.00E-96 | 98.47 | 347 | G |
| mutant GHu 1E^Pol^_K802G_ | 26 | FWD | JN391442.1  (GHu RNA1) | 6.00E-97 | 97.54 | 204 | G |
| mutant GHu 1E^Pol^_K802G_ | 26 | REV |  | 3.00E-100 | 99.49 | 203 | G |
| wildtype GHu | 26 | FWD |  | 8.00E-96 | 97.07 | 205 | K |
| wildtype GHu | 26 | REV |  | 5.00E-98 | 98.97 | 211 | K |

^a^Identity of amino acid residue 802 of the 1E^Pol^ protein confirmed via Sanger sequencing of RT-PCR amplicon (Osterbaan et al. 2019)

**Supplementary Table 2.** Primer sets used in RT-qPCR for grapevine fanleaf virus RNA1 using *Nicotiana benthamiana* FBOX as a reference gene.

| **Primer Name** | **Assay** | **Target** | **Sequence (5’-3’)** | **Reference** |
| --- | --- | --- | --- | --- |
| Nb-FBOX-F | RT-qPCR | *N. benthamiana* FBOX gene | ggcactcacaaacgtctatttc | Liu et al. 2012 |
| Nb-FBOX-R |  |  | acctgggaggcatcctgcttat |  |
| GFLV-RNA1-F |  | GFLV RNA1 sequence across coding regions of proteins 1D^Pro^-1E^Pol^ | cccaaaagtcatcgcaatgct | Osterbaan et al. 2019 |
| GFLV-RNA1-R |  |  | ggatcaggatatggaaagcac |  |
| GFLV-F13-1-F | RT-PCR | Sequence determinant region of GFLV RNA1 | tgttgggaccaaatcagaggaatg |  |
| GFLV-F13-1-R |  |  | gaaactgggttatttaaacttggt |  |
| GFLV-GHu-1-F |  |  | tgctaggaccaaatcagaggaacg |  |
| GFLV-GHu-1-R |  |  | aaacttggttatcccagtacca |  |

**Supplementary Table 3.** Root system architecture traits of *Nicotiana benthamiana* plants infected with four grapevine fanleaf virus strains (i.e., wildtypes GHu and F13, and mutants 1E^Pol*/Sd^_K802G_ and F13 1E^Pol*/Sd^_G802K_) at three time points following mechanical inoculation of leaves, i.e., 4-, 17-, and 26-days post-inoculation, as extracted from Rhizovision Explorer. Statistical comparison of means to mock control plants with significance thresholds of p-value < 0.05 (*); < 0.01 (**), < 0.001 (***), and <0.0001 (****) with Holm’s method adjustment. Plants at 4- and 17-dpi were compared through pairwise t-test for parametric data while plants at 26 dpi were compared through Dunn’s test for non-parametric data.

| days post inoculation | treatment | n | number of root tips | number of branch points | total root length (mm) | branching frequency | network area (mm^2) | average diameter (mm) | median diameter (mm) | perimeter (mm) | volume (mm^3) | surface area (mm^2) |
| --- | --- | --- | --- | --- | --- | --- | --- | --- | --- | --- | --- | --- |
| 4 dpi | mock control | 15 | 83 | 135 | 222.2 | 0.573 | 103.8 | 0.656 | 0.426 | 332.3 | 154.8 | 467.3 |
|  | wildtype F13 | 15 | 63 | 70*** | 147.2* | 0.461** | 84.7 | 0.786** | 0.467 | 228.9 | 140.5 | 370.0 |
|  | mutant F13 1E^Pol*/Sd^_G802K_ | 15 | 77 | 88* | 201.4 | 0.415*** | 116.7 | 0.806*** | 0.486 | 318.8 | 214.7 | 513.6 |
|  | wildtype GHu | 15 | 73 | 94 | 202.9 | 0.436*** | 119.2 | 0.822*** | 0.526* | 312.1 | 208.3 | 523.6 |
|  | mutant GHu 1E^Pol*/Sd^_K802G_ | 15 | 73 | 90* | 207.5 | 0.419*** | 118.3 | 0.809*** | 0.501 | 325.8 | 206.3 | 519.8 |
| 17 dpi | mock control | 14 | 164 | 754 | 1366.3 | 0.550 | 545.5 | 0.637 | 0.405 | 2099.0 | 922.8 | 2653.9 |
|  | wildtype F13 | 15 | 147 | 597 | 1216.9 | 0.486** | 556.9 | 0.720 | 0.466* | 1862.8 | 1000.6 | 2698.6 |
|  | mutant F13 1E^Pol*/Sd^_G802K_ | 15 | 110**** | 488*** | 1009.1** | 0.478** | 477.2 | 0.773*** | 0.494**** | 1505.2** | 997.3 | 2389.6 |
|  | wildtype GHu | 15 | 111**** | 460**** | 918.1*** | 0.494** | *434.5* | 0.777*** | 0.507**** | 1369.2**** | 881.7 | 2169.6 |
|  | mutant GHu 1E^Pol*/Sd^_K802G_ | 15 | 155 | 712 | 1287.5 | 0.541 | 554.7 | 0.702 | 0.447 | 1918.3 | 1060.4 | 2776.7 |
| 17 dpi | mock control | 14 | 188 | 460 | 936.8 | 0.482 | 526.0 | 0.893 | 0.584 | 1297.4 | 1354.7 | 2652.5 |
|  | wildtype F13 | 22 | 165 | 391 | 803.4 | 0.477 | 476.4 | 0.963 | 0.639 | 1076.2 | 1221.3 | 2432.0 |
|  | mutant F13 1E^Pol*/Sd^_G802K_ | 22 | 163 | 402 | 815.0 | 0.486 | 444.4 | 0.874 | 0.570 | 1127.4 | 1038.2 | 2230.0 |
|  | wildtype GHu | 23 | 132** | 351* | 738.7* | 0.460 | 414.9* | 0.911 | 0.606 | 1016.4 | 942.2 | 2076.0 |
|  | mutant GHu 1E^Pol*/Sd^_K802G_ | 19 | 163 | 368 | 779.5 | 0.462 | 463.3 | 0.974 | 0.643 | 1063.7 | 1126.7 | 2334.3 |
| 26 dpi | mock control | 26 | 257 | 550 | 1072.9 | 0.506 | 588.2 | 0.839 | 0.601 | 1501.4 | 1093.3 | 2804.2 |
|  | wildtype F13 | 14 | 266 | 559 | 1073.2 | 0.518 | 576.3 | 0.828 | 0.572 | 1497.6 | 1116.0 | 2795.2 |
|  | mutant F13 1E^Pol*/Sd^_G802K_ | 18 | 271 | 548 | 1066.7 | 0.511 | 631.3 | 0.942*** | 0.644 | 1443.3 | 1418.1* | 3130.5 |
|  | wildtype GHu | 15 | 193* | 416* | 819.5** | 0.500 | 461.4** | 0.884 | 0.642 | 1143.6 | 831.6 | 2184.2** |
|  | mutant GHu 1E^Pol*/Sd^_K802G_ | 7 | 295 | 578 | 1135.2 | 0.506 | 617.0 | 0.837 | 0.580 | 1584.9 | 1236.9 | 3019.7 |

**Supplementary Table 4.** Relative percentage change of root system architecture traits of *Nicotiana benthamiana* plants infected with four distinct grapevine fanleaf virus strains (i.e., wildtypes GHu and F13, and mutants GHu 1E^Pol*/Sd^_K802G_ and F13 1E^Pol*/Sd^_G802K_) compared with mock control at three time points (i.e., 4, 17 and 26 days post-inoculation) following mechanical inoculation of leaves. Statistical comparison of means to mock control plants with significance thresholds of p-value < 0.05 (*); < 0.01 (**), < 0.001 (***), and <0.0001 (****) with Holm’s method adjustment. Plants at 4- and 17-dpi were compared through pairwise t-test for parametric data while plants at 26 dpi were compared through Dunn’s test for non-parametric data.

| days post inoculation | treatment | n | number of root tips | number of branch points | total root length (mm) | branching frequency | network area (mm^2) | average diameter (mm) | median diameter (mm) | perimeter (mm) | volume (mm^3) | surface area (mm^2) |
| --- | --- | --- | --- | --- | --- | --- | --- | --- | --- | --- | --- | --- |
| 4 dpi | mock control | 15 | 0% | 0% | 0% | 0% | 0% | 0% | 0% | 0% | 0% | 0% |
|  | wildtype F13 | 15 | -23% | -48%*** | -34%* | -20%** | -18% | 20%** | 10% | -31% | -9% | -21% |
|  | mutant F13 1E^Pol*/Sd^_G802K_ | 15 | -7% | -35%* | -9% | -28%*** | 12% | 23%*** | 14% | -4% | 39% | 10% |
|  | wildtype GHu | 15 | -12% | -30% | -9% | -24%*** | 15% | 25%*** | *23% | -6% | 35% | 12% |
|  | mutant GHu 1E^Pol*/Sd^_K802G_ | 15 | -12% | -33%* | -7% | -27%*** | 14% | 23%*** | 18% | -2% | 33% | 11% |
| 17 dpi | mock control | 14 | 0% | 0% | 0% | 0% | 0% | 0% | 0% | 0% | 0% | 0% |
|  | wildtype F13 | 15 | -11% | -21% | -11% | -12%** | 2% | 13% | 15%* | -11% | 8% | 2% |
|  | mutant F13 1E^Pol*/Sd^_G802K_ | 15 | -33%**** | -35%*** | -26%** | -13%*** | -13% | 21%*** | 22%**** | -28%** | 8% | -10% |
|  | wildtype GHu | 15 | -32%**** | -39%**** | -33%*** | -10%** | -20%* | 22%*** | 25%**** | -35%**** | -4% | -18% |
|  | mutant GHu 1E^Pol*/Sd^_K802G_ | 15 | -5% | -6% | -6% | -2% | 2% | 10% | 10% | -9% | 15% | 5% |
| 17 dpi | mock control | 14 | 0% | 0% | 0% | 0% | 0% | 0% | 0% | 0% | 0% | 0% |
|  | wildtype F13 | 22 | -12% | -15% | -14% | -1% | -9% | 8% | 9% | -17% | -10% | -8% |
|  | mutant F13 1E^Pol*/Sd^_G802K_ | 22 | -13% | -13% | -13% | 1% | -16% | -2% | -2% | -13% | -23% | -16% |
|  | wildtype GHu | 23 | -30%** | -24%* | -21%* | -5% | -21%* | 2% | 4% | -22% | -30% | -22% |
|  | mutant GHu 1E^Pol*/Sd^_K802G_ | 19 | -13% | -20% | -17% | -4% | -12% | 9% | 10% | -18% | -17% | -12% |
| 26 dpi | mock control | 26 | 0% | 0% | 0% | 0% | 0% | 0% | 0% | 0% | 0% | 0% |
|  | wildtype F13 | 14 | 3% | 2% | 0% | 2% | -2% | -1% | -5% | 0% | 2% | 0% |
|  | mutant F13 1E^Pol*/Sd^_G802K_ | 18 | 5% | 0% | -1% | 1% | 7% | 12%*** | 7% | -4% | 30%* | 12% |
|  | wildtype GHu | 15 | -25%* | -24%* | -24%** | -1% | -22%** | 6% | 7% | -24% | -24% | -22%** |
|  | mutant GHu 1E^Pol*/Sd^_K802G_ | 7 | 15% | 5% | 6% | 0% | 5% | 0% | -3% | 6% | 13% | 8% |

**Supplementary Table 5.** Root system architecture (RSA) metrics of *Nicotiana benthamiana* at 4-, 17-, and 26 days post inoculation (dpi) with grapevine fanleaf virus (GFLV), organized by viral treatment group. Differences were observed largely between asymptomatic plants (mock control, wildtype GFLV-F13 and mutant GFLV-GHu 1E^Pol^_K802G_) and symptomatic plants (wildtype GFLV-GHu and mutant GFLV-F13 1E^Pol^_G802K_), where symptomatic plants had significantly less root tips, a larger root diameter, and decreased root branching points. Other metric averages include branching frequency, network area, median diameter, perimeter, root volume, and surface area. These data are also present on GitHub repository for ease of analysis with R Markdown files. **(.xlsx)**

**Sheet 1.** Summary of metrics from Rhizovision analysis.

**Sheet 2.** Metrics of data from Rhizovision and metadata measurements for plants inoculated with GFLV at 4 dpi.

**Sheet 3.** Metrics of data from Rhizovision and metadata measurements for plants inoculated with GFLV at 17 dpi.

**Sheet 4.** Metrics of data from Rhizovision and metadata measurements for plants inoculated with GFLV at 17 dpi, replicate 2.

**Sheet 5.** Metrics of data from Rhizovision and metadata measurements for plants inoculated with GFLV at 26 dpi.

**Supplementary Table 6.** Symptomology observations of *Nicotiana benthamiana* plants from infection of grapevine fanleaf virus strain wildtype GHu. Symptomology recordings represent three replicated experiments with confirmed infection status through DAS-ELISA, and strain specific infection status through RT-PCR amplicon sequencing for a subset of individuals. All experiments have at least three individuals sequenced and all individuals displaying symptoms of vein clearing were true to infection status. **(.xlsx)**

**3A.** Summary of grapevine fanleaf virus infected plants according to when they were first symptomatic, last symptomatic, and total days symptomatic.

**3B.** Binary rating (0=asymptomatic, 1=symptomatic) of three replicated experiments for vein-clearing symptoms of GFLV wildtype GHu infected plants from 0 to 17 days post inoculation.

**Supplementary Table 7.** Metrics corresponding to 3’RNA-Seq data acquisition, trimming (Trimmomatic), alignment/gene encoding product (HiSat2, FeatureCounts), and quality (FastQC) per sample. Alignment was performed against two *Nicotiana benthamiana* genomes at HiSat2, but only Niben101 (Bombarely et al. 2012) was utilized for performance. Viral counts were acquired through the “Nepo” curated database as performed previously (Osterbaan et al. 2021 and Roy et al. 2023) **(.xlsx)**.

**Supplementary Table 8.** All differentially abundant genes across all contrasts generated in DESeq2 analysis. (.**xlsx**)


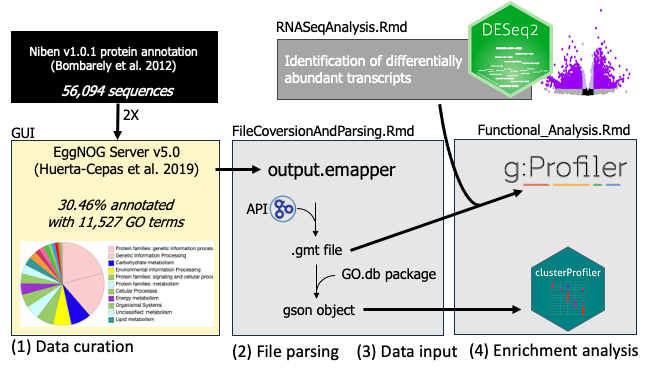


**Supplementary Figure 1.** Reannotation of the *Nicotiana benthamiana* genome from data curation to enrichment analysis at the terminal end of transcriptomic analysis. Over 50,000 protein encoding sequences were subject to the EggNOG server (Huerta-Cepas et al. 2019) where 30.46% of the sequences were annotated with 11,527 GO terms used once or more. (2) The output was cross-referenced to the GO API server in which a .gmt file was created for all genes annotated as protein. Further, the .gmt file was parsed to include term2name and gsid2gene as required for the creation of a gson object by the use of the GO.db package. (3) RNA-Seq differential abundance analysis was performed with DESeq2 and the resulting output of gene lists were used for (4) enrichment analysis. The curated .gmt file and gson object were used as input for use in gProfiler2 and clusterProfiler, respectively. All code utilized in steps 2, 3, and 4 are found in as R markdown files in the supplementary information.


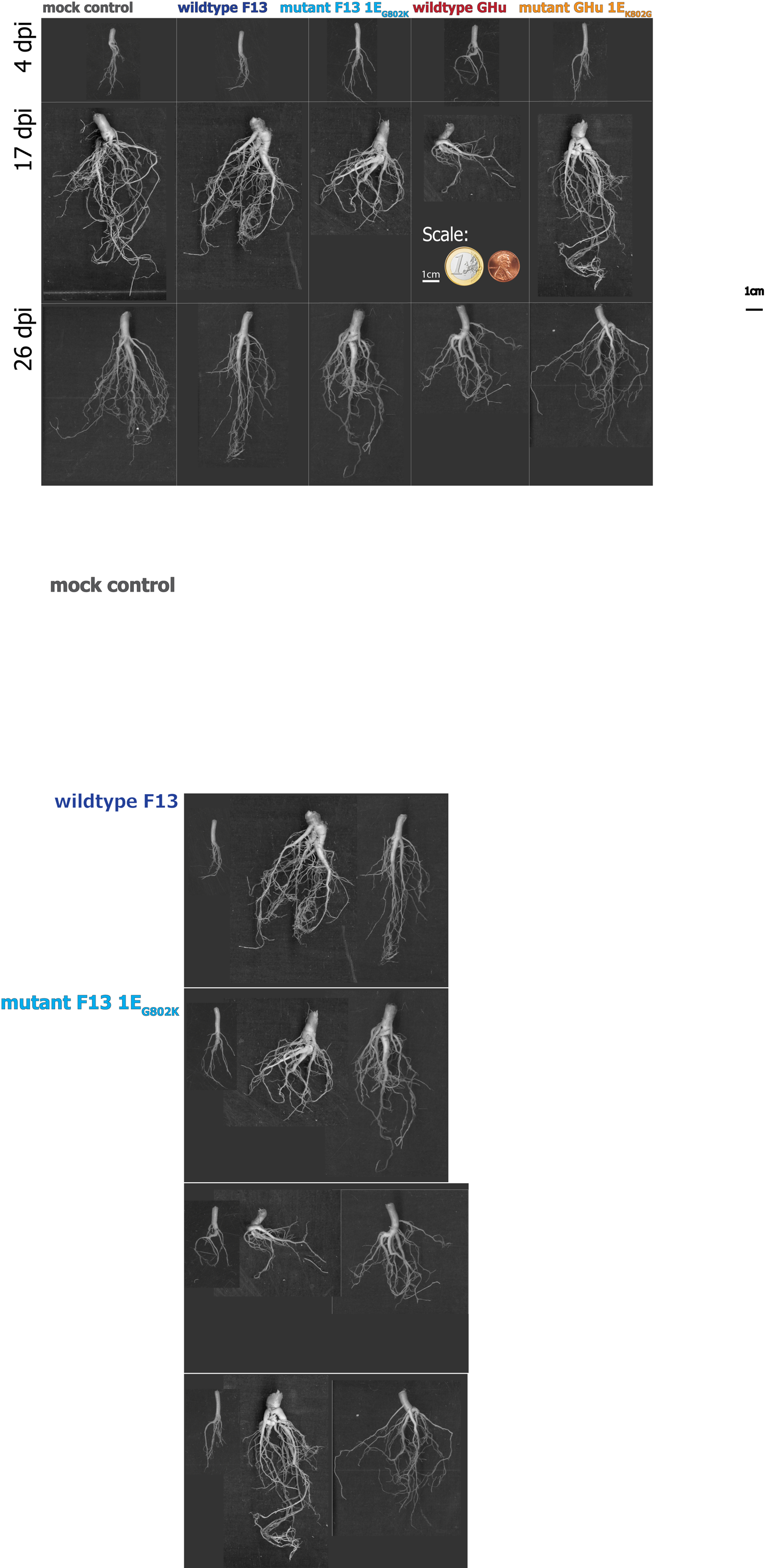


**Supplementary Figure 2.** Representative root phenotypes of *Nicotiana benthamiana* upon differential grapevine fanleaf virus strain (wildtype GFLV-F13 and wildtype GFLV-GHu) and mutant (GFLV-F13 1E^Pol^_G802K_ and GFLV-GHu 1E^Pol^_K802G_) infections at four-, 17-, and 26 days post inoculation (dpi). All roots are scaled to 1 cm through pixels per square inch and using ImageJ, with reference coins for visualization purposes.


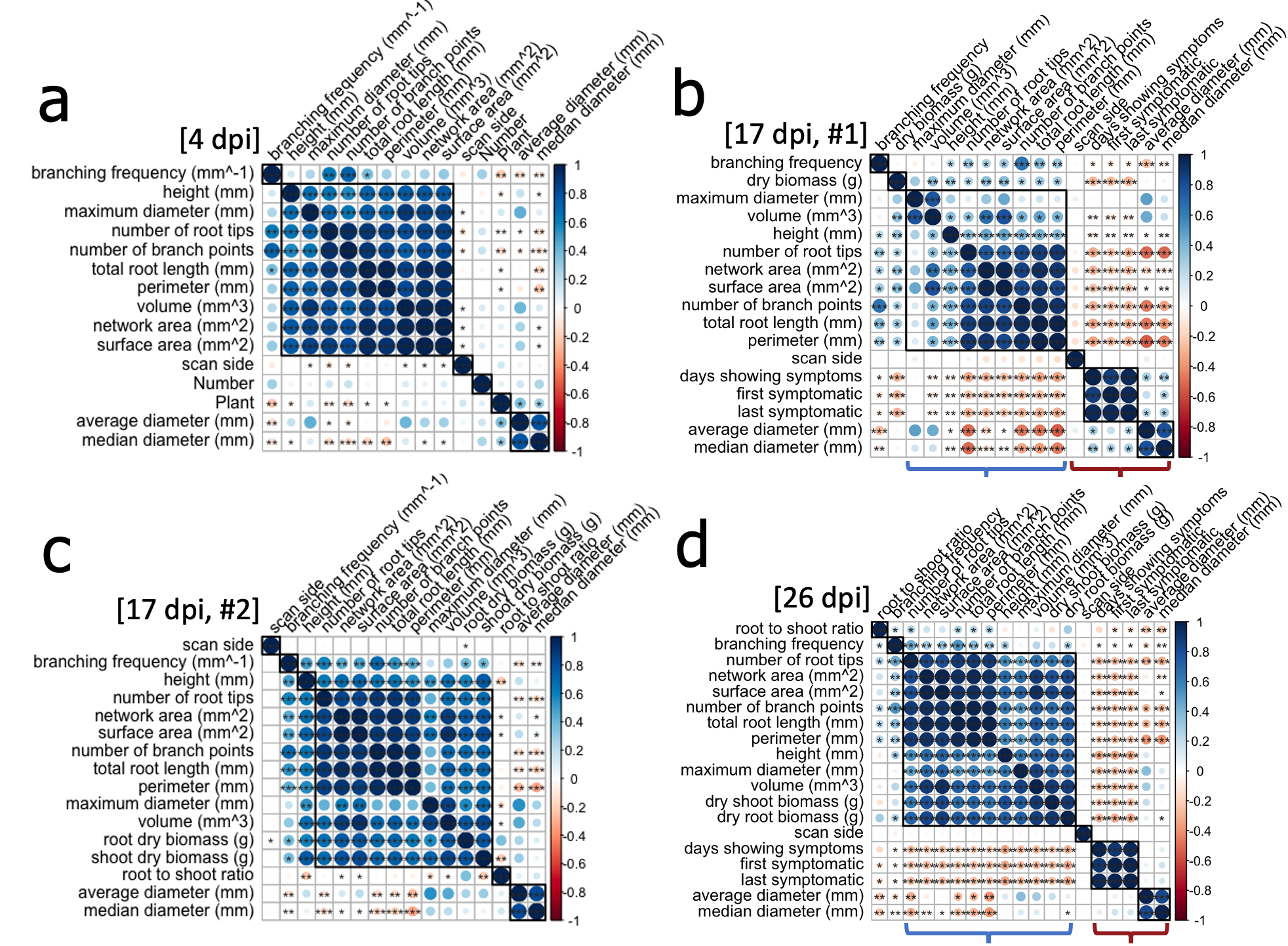


**Supplementary Figure 3.** Correlation matrices of acquired root phenotypic data for *Nicotiana benthamiana* infected with grapevine fanleaf virus (GFLV) **(a)** Correlation of root system and total plant metrics at 4 days post inoculation (dpi), **(b)** 17 dpi, **(c)** 17 dpi replicate 2, and **(d)** 26 dpi. Positively and negatively correlated metrics are denoted in blue and red, respectively, with color opaqueness and size increasing with larger absolute correlation. Significant correlation of traits is denoted by p-value as asterisks with increasing intensity (* *P* < 0.05, ** *P* < 0.01, *** *P* < 0.001). Hierarchal clustering method was employed through corrplot in RStudio, with square groupings assigned through clustering (rect = 6). Two ‘phene’ groups were assigned based on large, significant positive or negative correlation with each other as phene 1 (blue bracket) and phene 2 (red bracket). These phene group traits were consistently observed as highly correlated upon GFLV infection in *Nicotiana benthamiana* for all time points.


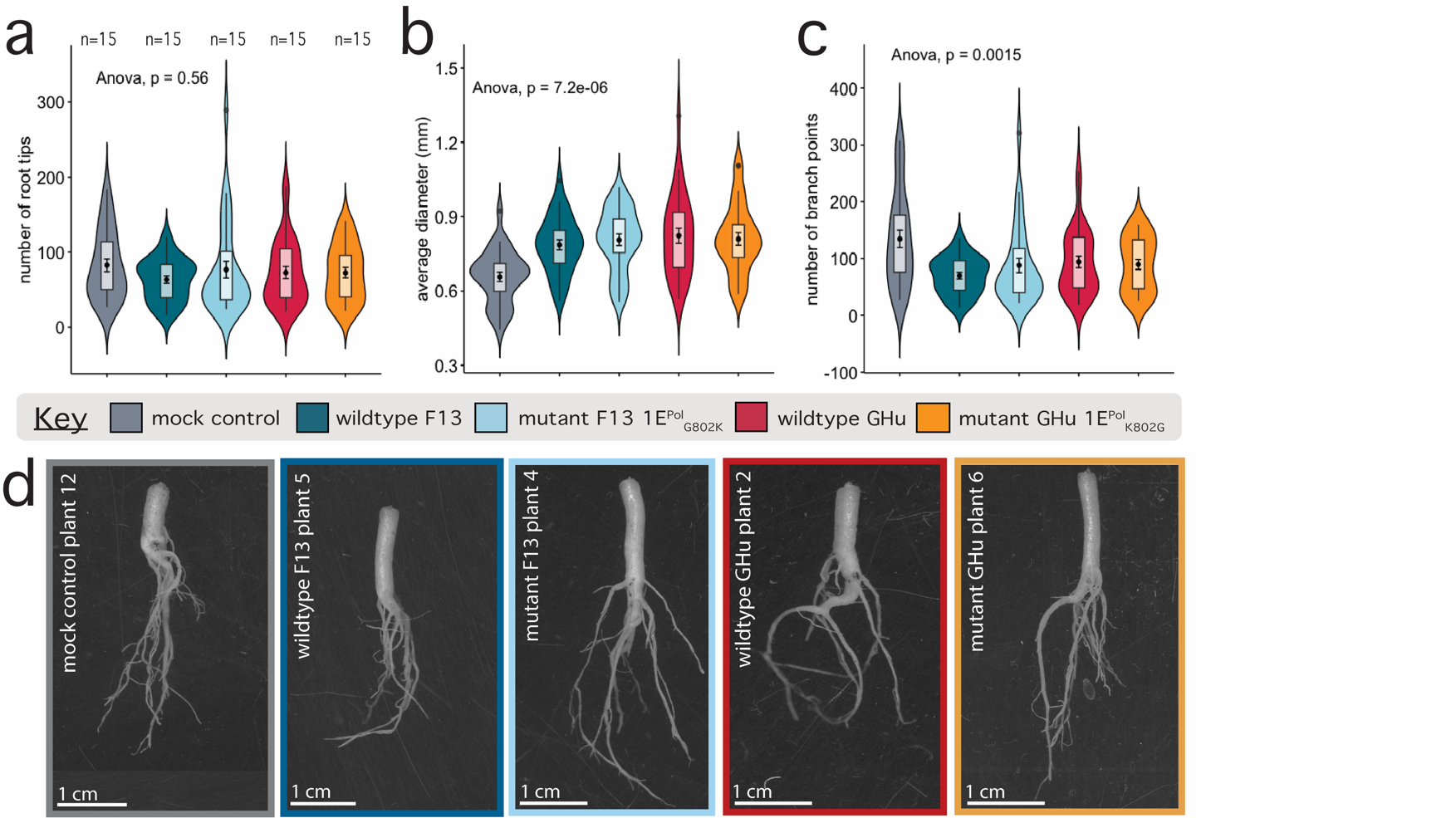

**Supplementary Figure 4.** Root system architecture traits for *Nicotiana* *benthamiana* infected with four strains of grapevine fanleaf virus at 4 days post inoculation (dpi). **(a)** Number of root tips per plant, **(b)** average diameter in millimeters, and **(c)** number of branch points plotted per viral treatment group. Distribution of each continuous value is plotted along the y-axis as a violin plot with boxplot within, showing additional mean with standard error (central point with error bars). Pairwise Wilcox test results are displayed as significance values (*P* < 0.05=*, *P* < 0.01=**, *P* < 0.001=***, *P* < 0.0001=****). Number of samples per treatment are displayed once above panel (a). **(d)** Representative images of mean phenotype observed between treatments, border color is indicative of treatment as in plots A, B, and C. White bar displayed in bottom left corner of each image is 1.0 centimeters, calculated in ImageJ software (v1.8.0.172).


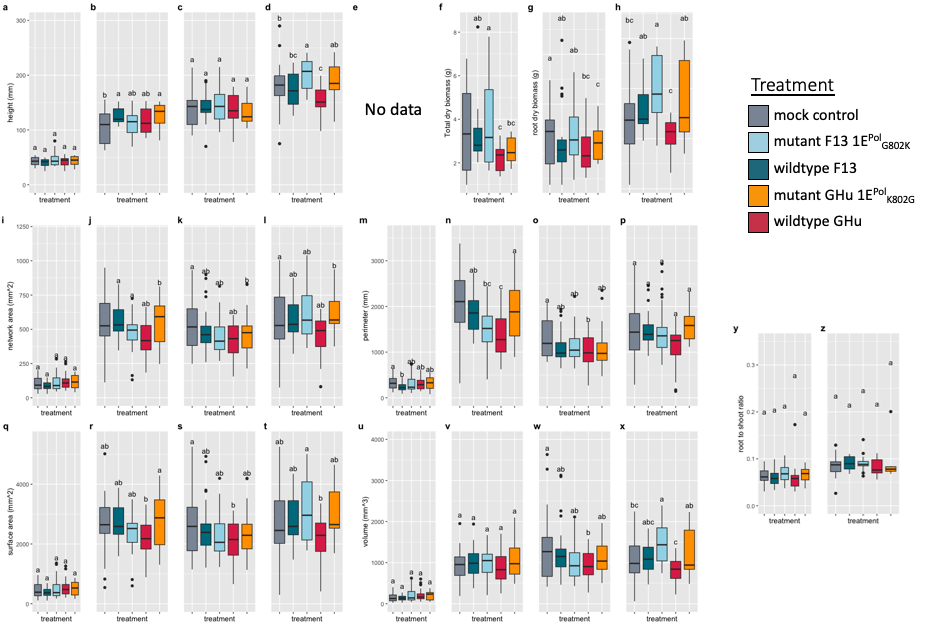


**Supplementary Figure 5.** Additional traits of *Nicotiana benthamiana* infected with grapevine fanleaf virus (GFLV) strains at various days post inoculation (dpi). Height of plant from soil-barrier line in millimeters at (a) 4 dpi, (b) 17 dpi, (c) 17 dpi, and (d) 26 dpi show more statistically validated dissimilarities later in infection. Biomass tissue in grams at (e) 4 dpi with no data, (f) 17 dpi for total dry biomass, and dry root biomass for (g) 17 dpi (h) 26 dpi show significant differences for several treatments. The network area of root system in square millimeters at (i) 4 dpi, (j) 17 dpi, (k) 17 dpi, and (l) 26 dpi show minor separation by treatment. The perimeter of root system in millimeters at (m) 4 dpi, (n) 17 dpi, (o) 17 dpi, and (p) 26 dpi show transient significant differences. The calculated surface area in square millimeters at (q) 4 dpi, (r) 17 dpi, (s) 17 dpi, and (t) 26 dpi show separation of wildtype GHu starting at 17 dpi. The projected volume of root system in cubed millimeters per viral treatment group at (u) 4 dpi, (v) 17 dpi, (w) 17 dpi, and (x) 26 dpi show again sustained differences for wildtype GHu according to Tukey tests. No significant differences were observed for root to shoot ratio at 17 dpi (y) or 26 dpi (z). Distribution of each continuous value is plotted along the y-axis as a box plot. Tukey’s honest significant difference test was performed across all treatments to assign Tukey letters and display above each treatment.

**
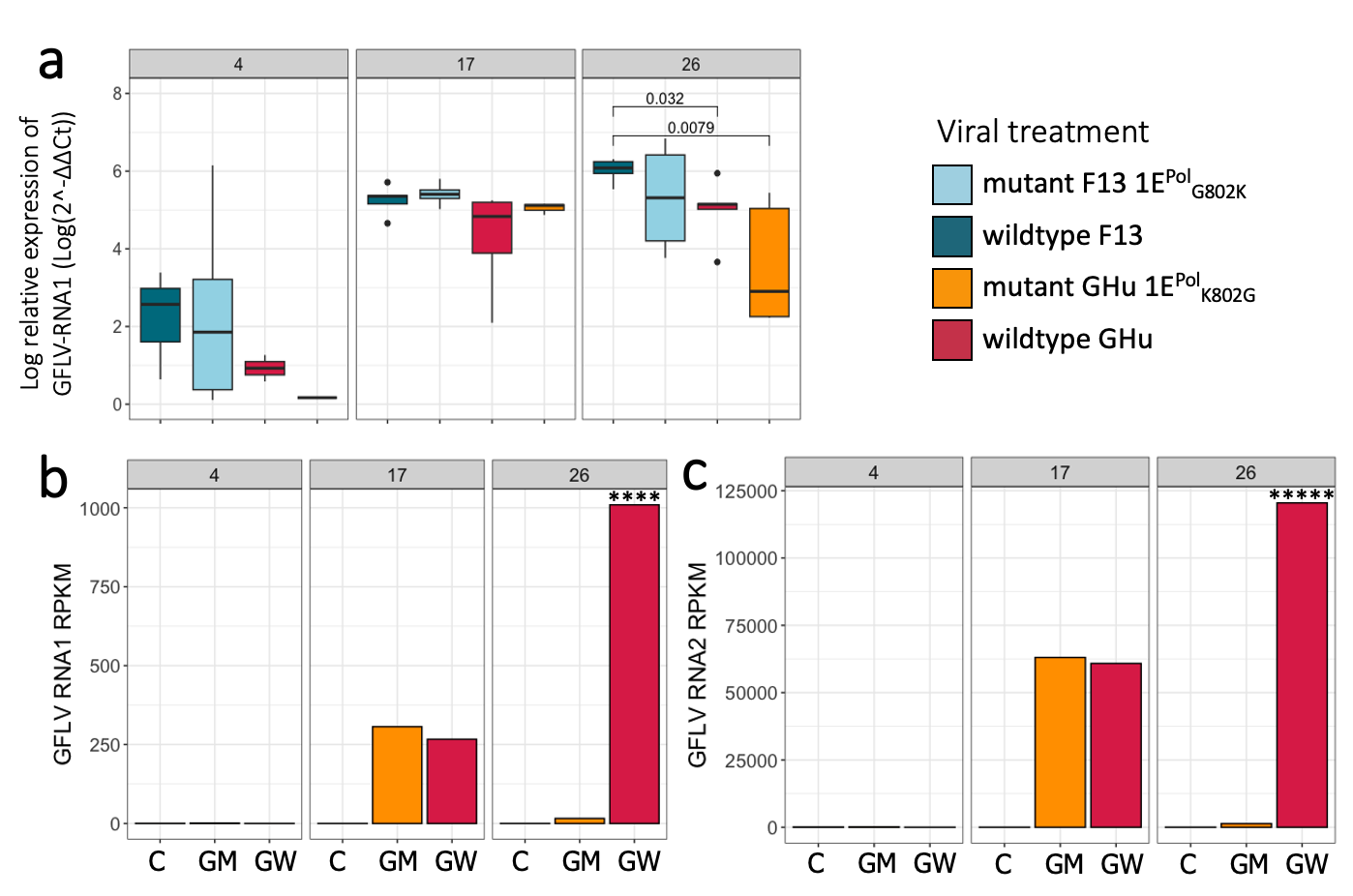
**

**Supplementary Figure 6.** Quantitative real-time polymerase chain reaction- and 3’RNA-sequencing-based detection of grapevine fanleaf virus (GFLV) from Nicotiana benthamiana root tissue samples reveal differences in viral titer by strain late in infection. **(a)** Treatment-wise comparison at 4-, 17-, and 26 days post inoculation (dpi) show treatments do not vary in viral RNA1 titer by Wilcoxon geometric comparison of means except for wildtype GFLV-F13 strain (*P* ≤ 0.032) at 26 dpi when compared to either GFLV-GHu strain. **(b)** Normalized GFLV RNA1 reads per kilobase million (RPKM) extracted from RNA-Seq data reveal significantly more abundant reads of wildtype GFLV-GHu when compared to mutant GFLV-GHu 1E^Pol^_K802G_ at 26 dpi (Tukey’s HSD, *P* = 0.00014), and **(c)** an increased retrieval amount of GFLV-RNA2 reveal a similar comparison at 26 dpi (Tukey’s HSD, *P* = 0.0000008).


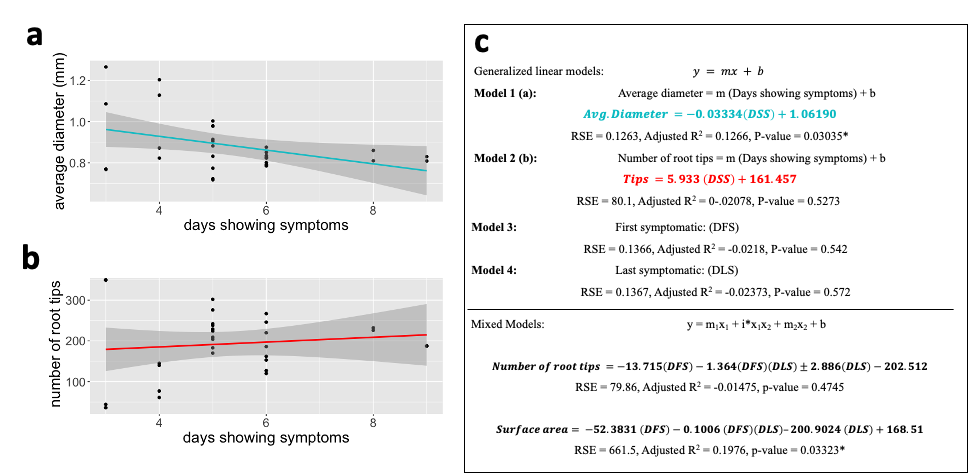


**Supplementary Figure 7**. Linear model generation for *Nicotiana benthamiana* infected with wildtype grapevine fanleaf virus strain GHu at 26 days post inoculation (dpi) return mostly insignificant predictions of above and below ground phenotypes. **(a)** Using the number of days showing symptoms, a predictive linear model was generated to reflect the average diameter on the y-axis. This model retained a significant p-value however other metrics were of low quality (RSE and adjusted R^2^ values). **(b)** In a similar way, days showing symptoms were plotted against the number of root tips, however retained a poor p-value (0.5273) and was inconsistent with previous findings. **(c)** Linear model formulaic construction considered several biologically important parameters, however few had acceptable metrics for a complete model to be considered at this time point.

**
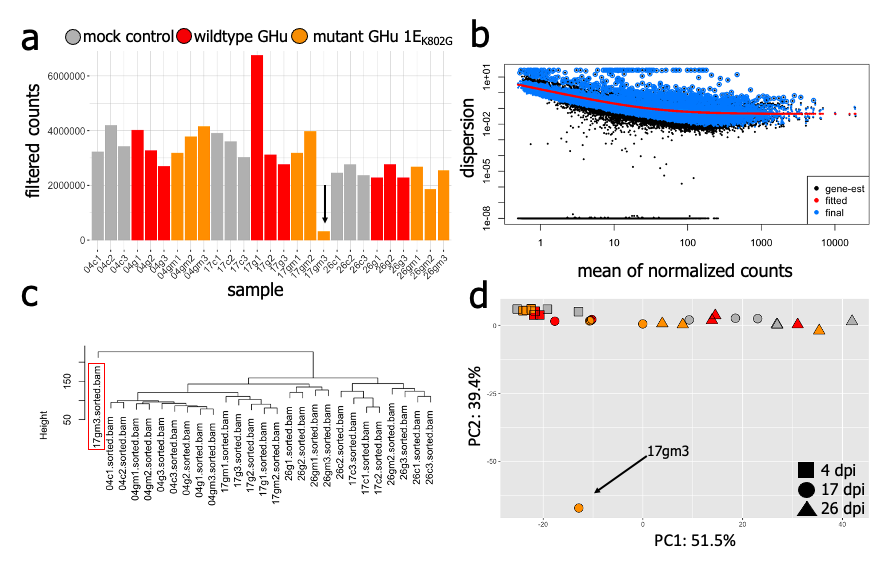
**

**Supplementary Figure 8.** Primary analysis of input RNA-Seq data shows presence of outlier of mutant GFLV-GHu 1E^Pol^_K802G_ at 17 dpi due to low counts acquired. **(a)** Filtered reads per sample show many samples around 3,000,000 reads except for ‘17g1’ and ‘17gm3’, towering red bar and small orange bar indicated by arrow, respectively. Treatments are colored gray for mock control, red for wildtype GFLV-GHu treated plants, and orange for mutant GFLV-GHu 1E^Pol^_K802G_ treated plants. Time scale from 4-, 17-, and 26-days post inoculation (dpi) are visualized from left to right in groups of nine samples. **(b)** Dispersion estimates output of DESeq2 package analysis of RNA-Seq reads. Many reads diverge from fitted line in red, which may correspond to dissonance of samples with varying read number. **(c)** Hierarchal clustering (McQuitty method) groups samples by time except for outlier of ‘17gm3’, indicated by red box, post-normalization. **(d)** Principal component analysis of samples post-normalization through ‘vsd’ method native to DESeq2 package shows outlier ‘17gm3’ separating from all other samples.

**
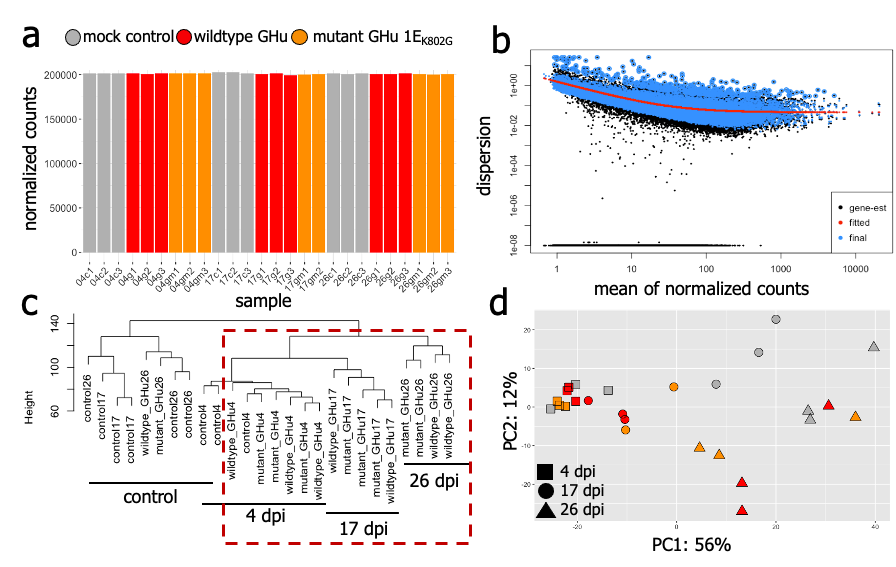
**

**Supplementary Figure 9.** Analysis of input RNA-Seq data shows grouping of viral treated individuals and acceptable input for differential abundance testing after removal of outlier. **(a)** Bar plot depiction of total counts after filtering and normalization per sample. Left to right colored gray, red, and orange are treatments mock control, wildtype GHu, and mutant GHu 1E^Pol^_K802G_ , by times of 4-, 17-, and 26 dpi. **(b)** Dispersion estimates of counts plot shows fitted line with decreased final values placed above or below y-axis bounds **(c)** Hierarchal clustering of samples through McQuitty method after removal of outlier (wildtype GHu #3). Virally infected samples at 4-, 17-, and 26 dpi group to the right while mock inoculated control plants group together on the left. **(d)** Principal component analysis of all samples shows distinct separation by time through PC1 [56%], and additional but not complete separation of treatment through PC2 [12%].

**Supplementary Figure 10.** Experiment wide gene set enrichment analysis (GSEA) shows small overlaps of treatment-wise comparisons of overrepresentation and many unique attributes over time. Input parameters required differentially expressed genes with p-value<0.05 and |Log2FoldChange|>0.75.


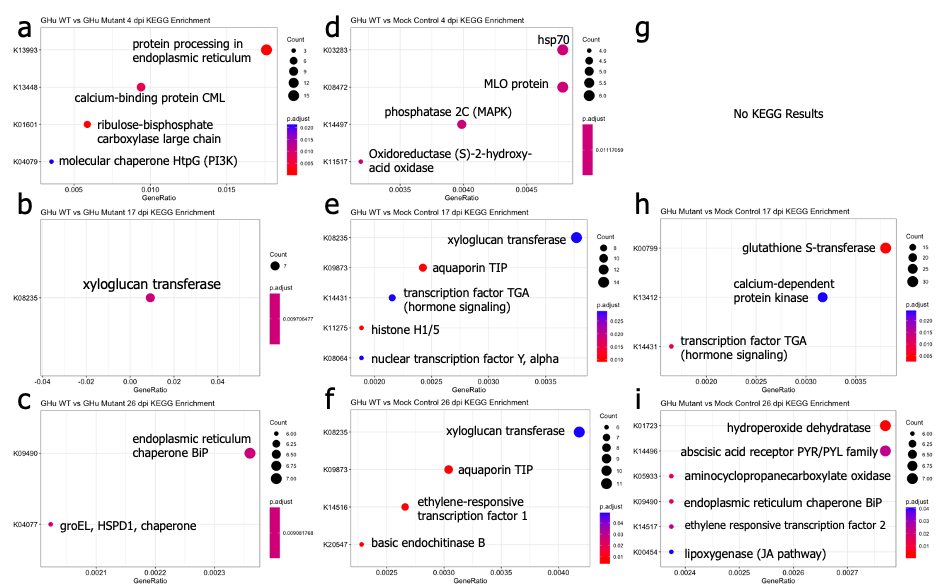


**Supplementary Figure 11.** Enrichment results of differentially abundant host genes through Kyoto Encyclopedia of Genes and Genomes (KEGG) analysis of grapevine fanleaf virus infected *Nicotiana benthamiana*. Each panel is representative of an individual treatment contrast per time point in which differentially abundant genes were tested for overrepresentation of KEGG terms. Manual labels were curated to each data point according to the corresponding KEGG identifier. From blue to red, the color shows increased significance of enrichment while the size of the datapoint is relative to the number of genes in that category. The distance from the x-axis intercept is relative of the gene ratio or the relative amount of genes found in that category to the total number of genes subject to the test. The contrast of wildtype GHu against mutant GHu 1E^Pol^_K802G_ is observed at **(a)** 4 days post inoculation (dpi), **(b)** 17 dpi, and **(c)** 26 dpi. Similarly, the contrast of wildtype GHu versus mock inoculated control plants are shown for overrepresented KEGG terms at **(d)** 4 dpi, **(e)** 17 dpi, and **(f)** 26 dpi. **(g)** No significant KEGG terms returned at 4 dpi for the contrast of mutant GHu against mock control plants, however several terms were significant in this contrast at **(h)** 17dpi and **(i)** 26 dpi.
